# Motor Preparatory Activity in Posterior Parietal Cortex is Modulated by Subjective Absolute Value

**DOI:** 10.1371/journal.pbio.1000444

**Published:** 2010-08-03

**Authors:** Asha Iyer, Axel Lindner, Igor Kagan, Richard A. Andersen

**Affiliations:** Division of Biology, California Institute of Technology, Pasadena, California, United States of America; National Institutes of Mental Health, United States of America

## Abstract

For optimal response selection, the consequences associated with behavioral success or failure must be appraised. To determine how monetary consequences influence the neural representations of motor preparation, human brain activity was scanned with fMRI while subjects performed a complex spatial visuomotor task. At the beginning of each trial, reward context cues indicated the potential gain and loss imposed for correct or incorrect trial completion. FMRI-activity in canonical reward structures reflected the expected value related to the context. In contrast, motor preparatory activity in posterior parietal and premotor cortex peaked in high “absolute value” (high gain or loss) conditions: being highest for large gains in subjects who believed they performed well while being highest for large losses in those who believed they performed poorly. These results suggest that the neural activity preceding goal-directed actions incorporates the absolute value of that action, predicated upon subjective, rather than objective, estimates of one's performance.

## Abstract 2

Cortical motor planning is shaped by “subjective absolute value”: planning activity is strongly enhanced for large expected gains in subjects who believe they perform well; conversely, activity is higher for large expected losses in subjects who think they perform poorly.

## Author Summary

The expected outcome of voluntary actions profoundly shapes human decision making. For instance, expected monetary reward and punishment are powerful modulators of human behavior. Yet how these factors influence brain activity responsible for the preparation of such behavior is not fully understood. This is especially true for demanding tasks, in which the outcome—e.g. reward versus punishment—critically depends on the accuracy of actions. In our human fMRI study we investigated brain activity in specific cortical areas that are related to the planning of voluntary behavior. We show that planning activity in these areas is strongly influenced by the expected monetary gain or loss that subjects associated with their performance in a demanding motor task. Planning activity was highest for large expected gains in subjects who believed that they performed well; conversely, activity was highest for large expected losses in subjects who thought that they performed poorly. This pattern of planning activity was best described by a model which we refer to as the “subjective absolute value model.” We suggest that absolute value signals in motor planning areas can be used to mobilize motor resources in behaviorally relevant situations—both to maximize gains and to avoid losses.

## Introduction

The selection of one amongst a repertoire of potential behavioral responses entails the articulation of both an appropriate goal and the means to achieve that goal. In a natural context, however, a plan of action rarely guarantees a specific outcome. Most actions carry with them a certain probability of success or failure, and these successes and failures engender certain consequences. Thus to discern an optimal course of action, the expected consequences of actions—their possible outcomes and contingencies—must be assessed.

Functional imaging studies in humans have extensively investigated areas differentially responsive to various aspects of choice [1]–[4], anticipation [5]–[8], and receipt of monetary gains and losses [9]–[14]. Predominantly, these inquiries have emphasized subcortical and prefrontal cortical regions, speculating on their role in an array of tasks from facilitating appropriate approach or avoidance behavior to monitoring outcomes in order to adjust future strategies.

From this wealth of findings, knowledge has been gleaned as to how rewards associated with stimuli are processed and exploited to guide behavior. However, these studies shed considerably less light on whether and how rewards consequent of response execution mold motor-preparatory activity in the areas engaged in transforming sensory inputs into preparatory signals preceding motor events. Most previous experiments have passively presented cues and outcomes, demanding no instrumental response on the part of the subjects to obtain rewards [11],[15]–[18]. Even in paradigms mandating movements—either as tools to maintain vigilance or signal choice [3],[6],[7],[19]–[21], or specifically to investigate instrumental action-reward contingencies—the required responses were comparatively easy, thus not prompting substantial motor preparation [22]–[26].

In recent years, macaque electrophysiological experiments have begun dissecting the influence of reward contingencies on the process of action selection and preparation. These investigations have identified reward-related factors that bias neural activity in motor preparatory frontal and posterior parietal areas, which may in turn dispose the animal's selection of which movement to execute. Firing rates in lateral intraparietal area (LIP), the region in macaque posterior parietal cortex involved in encoding oculomotor action plans [27], have been shown to be correlated with the weight of sensory input indicating which saccade target is rewarded [28], the log likelihood that a given eye movement will result in a reward [29], the magnitude and probability of reward associated with a saccade target [30],[31], and the relative desirability of a saccade with respect to other possible saccade options [32]. Information about the preference and magnitude of rewards for reach targets has been decoded from an adjacent parietal region involved in reaching [33],[34], and recordings from premotor cortex imply that the motivation to choose and acquire a saccade target may shape neural responses as well [35].

While these investigations proffer insight into reward-modulated motor preparatory activity, they often employed behavioral tasks that were rather undemanding and simple, as in most human studies examining reward. Conversely, many real-life goal-directed actions necessitate greater cognitive exertion, demanding effort at mnemonic, preparatory, and/or execution stages. This complexity generates uncertainty and variability in behavioral outcomes. The prediction and evaluation of these potential behavioral outcomes under difficult task conditions clearly modulates the neural representations of reward and punishment [7],[36],[37] and, moreover, allows optimizing motor responses [38]. Yet little is known about *where* and *how* outcome-related parameters might influence neural activity subserving action preparation. In addition, the corpus of previous studies on reward-modulated motor preparatory activity largely assigned absence of reward rather than explicit punishment as the cost of failure, impeding distinctions between factors associated with a given action (but see [35]). Without explicit penalties, variables such as value, incentive (i.e. aversion to punishment or the expectation of reward), and internal motivation would likely change in step. Finally, as simple movements render the likelihood of success high and the ability to gauge one's own performance straightforward, the effects of subjects' *subjective* appraisal of outcomes as opposed to the actual *objective* probability of outcomes cannot easily be disentangled. Thus, it is difficult to infer from previous work how these different outcome-related parameters impinge upon the neural representations of complex behaviors required in everyday life.

The goal of this study was to ascertain whether and, if so, how expected consequences of complex actions, dependent on human subjects' performance, modulate activity of neural substrates engaged in action preparation. Using event-related fMRI, we investigated effects of expected monetary reward or punishment in cerebral areas recruited in a challenging spatial delayed-response task. To impose consequences for success and failure, trials were associated with variable monetary gain-loss contexts, stipulating at the beginning of the trial the amount the subject would gain if she/he performed the task correctly and lose otherwise. Every trial instructed one correct response, so subjects unequivocally understood the appropriate action to garner success and maximize reward. Therefore, unlike most prior studies, sizable uncertainty in anticipation of reward or punishment stemmed entirely from the subject's ability to successfully prepare and implement the pre-cued motor response.

This study reports that the profile of motor preparatory activity throughout several task-relevant regions manifested modulation due to the gain-loss contexts. Specifically, signal time-courses of regions in posterior parietal and premotor cortex reflected the action's *absolute value* in the delay period preceding the response. Moreover, these areas revealed a cognitive, framing effect, responding as dictated by *subjective* estimates of success rather than subjects' objective performance: motor preparatory activity was more pronounced in higher gain conditions whenever a subject thought that she/he performs well, whereas preparatory activity was increased in higher loss conditions whenever a subject thought that she/he performs poorly.

## Results

**Figure 1:**
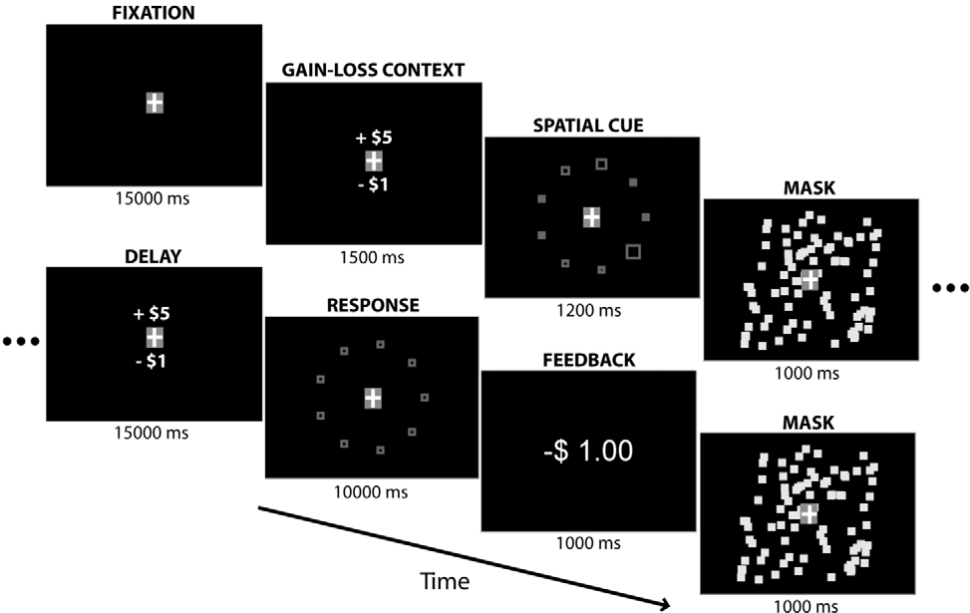
Experimental design. After initial baseline fixation, the gain-loss contingencies for the trial were displayed, followed by a brief presentation of spatial cues specifying the required movements for the trial. After a long delay, subjects performed a speeded motor response, and received immediate feedback (gain or loss) based on their performance.

The principal events of interest in the task included: (1) the presentation of the gain-loss context cue, followed by the spatial cues; (2) the delay period interposed between visual cue presentation and the motor response; (3) the execution of the motor response; and (4) feedback indicating the gain or loss acquired in a particular trial, contingent on the correct (gain) or incorrect (loss) response (Figure 1). By imposing a long delay between instructive visual cues and the contingent motor response, this task structure permits delineation between neuronal contributions due to sensory, motor, and intervening preparatory processes.

To make a response, subjects operated a trackball with their right index finger to guide a cursor sequentially to five remembered out of nine possible target locations, in the exact order in which they were previously cued. Subjects were allowed a limited time in which to complete their motor responses, prompting them to prepare movements in advance. Brief cue presentation, high planning load and constrained response time made successful trial completion difficult. Therefore, subjects trained extensively on the task before scanning. This training helped to minimize learning effects and to stabilize performance during the experimental session, promoting stable expectations of action outcomes throughout the task (see below).

### Model Predictions for Different Gain-Loss Contexts

**Figure 2:**
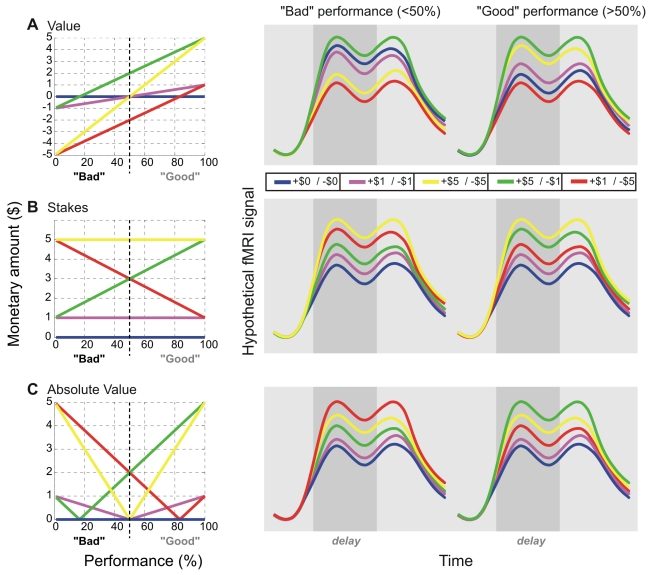
Explanatory models. Model predictions and hypothetical BOLD responses in motor-preparatory ROIs encoding value (A), stakes (B), and absolute value (C). For the purpose of illustration, three-component response profiles (cue peak, sustained delay, and response peak)—typically observed in motor preparatory regions—are depicted. The delay period is shaded in gray. Examples are given for both good (>50%) and bad performance (<50%).

The reward contexts comprised five combinations of potential gains and losses: $0/−$0, $1/−$1, $1/−$5, $5/−$1, and $5/−$5. These combinations enabled predictions as to the hypothetical modulation of neural signals due to various parameters of the expected action outcome for different performance levels. Figure 2 illustrates the models based on three such parameters, and corresponding predictions for fMRI activity in motor preparatory areas, averaged over “good” (>50%) and “bad” (<50%) performances:

1. First, possible gains and losses may be reflected in the prospective monetary return or “value” of an action. Value is calculated as the sum of two products—likelihood of success (i.e. performance) times gain plus likelihood of failure (i.e. 1−performance) times loss:
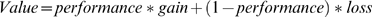
The value model predicts the highest and lowest BOLD-signal amplitudes for the “asymmetric,” higher gain ($5/−$1) and the higher loss condition ($1/−$5), respectively (Figure 2A). For bad performance BOLD-amplitudes increase from the $5/−$5 to the $1/−$1 to the $0/−$0 context, while for good performance this order reverses.
2. Second, possible gains and losses may be encoded in a manner that reflects the behavioral import of an action, either through acquiring a reward *or* through avoiding a loss. Indeed, avoiding a loss may in itself be rewarding, and a possibility of a loss may be as effective for mobilizing action preparation as a possibility of a prospective gain. Thus, potential gains and losses could both contribute to the appraisal of the action—either separately (i) or in combination (ii):
  - Separate contributions of potential gains and losses are captured by the “stakes” model (note the absolute value of the *loss* term):
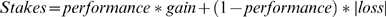
Following this model, at any level of performance the greatest modulation would be observed in the high gain/high loss context ($5/−$5); the smallest in the no gain/no loss context ($0/−$0) (Figure 2B). The ordering of the remaining contexts is dictated by performance. Note that on average (i.e. at 50% performance), the stakes model resembles the risk associated with an action since the stakes scales with the variance of the expected outcome.
  - Alternatively, a combined contribution of potential gains and losses to the appraisal of an action is captured by the “absolute value” model. In this model the expected gains and losses are summed as in the value model. Yet the result, either positive or negative, would equally translate into the representation of an action's import as captured by its absolute value (note that the term “absolute” here refers to the mathematical notion of “modulus”):
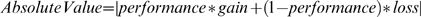
As contrasted to the stakes model, the absolute value model predicts the highest BOLD-amplitudes for “asymmetric” contexts: in the higher gain condition ($5/−$1) for subjects performing well, and in the higher loss condition ($1/−$5) for subjects performing poorly. In other words, the absolute value model highlights the possibility to obtain a reward in good performers whereas it stresses the chance to avoid a punishment in poor performers.

### Behavioral Results

**Figure 3:**
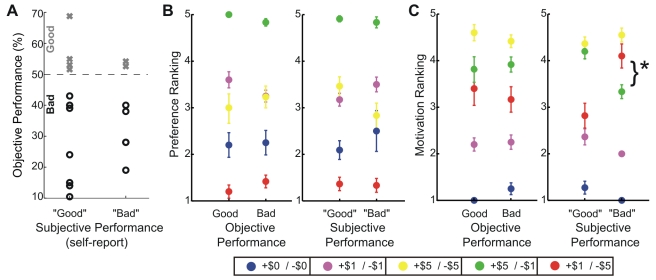
Subjects' performance and preferences. (A) Actual (objective) versus self-reported (subjective) performance. “Subjective good” subjects assumed net winning money; “subjective bad” subjects assumed the converse. For comparison, “x”s are subjects who actually won money; “o”s, subjects who lost money. (B) Subjects' average preference rankings ([1] low; [5] high) of gain-loss contexts, grouped by objective (left graph) and subjective (right graph) performance. (C) Subjects' average motivation rankings for varying gain-loss contexts, grouped by objective (left) and subjective (right) performance. Error bars reflect the standard error (SE) of the mean.

The 17 subjects who participated in this study achieved drastically different levels of performance, ranging from 10% to 70% correct responses (Figure 3A). However, performance levels across the five gain-loss contexts were indistinguishable (Friedman's ANOVA: X2 (4,64)  =  0.82, *p*  =  0.94). To assess if performance changed throughout the scanning session, trials were evenly divided into six successive blocks. No significant differences in success rates across blocks of trials emerged (Friedman's ANOVA: X2(2,32)  =  0.22, *p*  =  0.89), indicating that no learning occurred during the course of the fMRI experiment.

Across gain-loss contexts, reaction times were indistinguishable (Friedmans ANOVA: X2(4,960)  =  2.36, p  =  0.67). Total movement time to complete responses was shortest for the $5/−$1 condition. Yet this trend did not reach significance (Friedmans ANOVA: X2(4,960)  =  4.68, *p*  =  0.32). From these observations, individual subjects' behavioral measures show no significant disparities across conditions, yielding a relatively fixed probability of success for each subject during the experimental session.

### Subjective Reports

Upon completion of the scanning session, but prior to receiving any feedback about their overall performance and net winnings, all subjects completed a questionnaire: first, 16 of 17 subjects claimed to pay attention to the presented gain-loss contexts; all subjects reported investing maximal effort on all trials, independent of the gain-loss context, as instructed (see Experimental Procedures). Based on feedback received at the end of each trial, subjects also estimated whether they had net won money, net lost money, or broke even. Given the task structure and the fact that performance did not differ between conditions, net winning required >50% performance on trials resulting in earning increments/decrements; net losing required <50% performance on these trials. Figure 3A portrays the relationship between perceived (subjective) task winnings and subjects' average (objective) performance across all trials. The subjective “good” group claimed a net gain based on their performance during the task (*n*  =  11); the “bad” group claimed net losses (*n*  =  6). For comparison, subjects denoted by “x” actually net won money (*n*  =  6) during the scanning session, and those by “o” net lost (*n*  =  11). Note that the objective and subjective performances were uncorrelated (Behrens-Fisher two-sampled *t*-test comparing actual performance of the subjective good versus subjective bad groups: *p*  =  0.70). Because of this dichotomy, we will present all further results as a function of both objective and subjective performance estimates.

Additionally, subjects rated the gain-loss contexts in terms of their motivation during and their preference for related trials. Figure 3B depicts the mean preference: on the left, the ratings for the objective good versus objective bad subjects, and on the right, subjective good versus subjective bad subjects. Intuitively, these ratings should parallel the value associated with the gain-loss contexts. Accordingly, subjects in all groups most preferred the high-gain/low-loss context ($5/−$1), and least preferred the converse, low-gain/high-loss context ($1/−$5). Between the objective good and bad groups, no significant differences existed in the ratings of these and all remaining contexts (two-way mixed-design ANOVA: context [repeated measure]: *F*(4,60)  =  31.0, *p* < 0.05; group: *F*(1,15)  =  0, *p*  =  1.0; group *x* context: *F*(4,60)  =  0.3, *p*  =  0.89). A similar picture surfaced for the subjective good and bad groups (two-way mixed-design ANOVA: context [repeated measure]: *F*(4,60)  =  32.7, *p* < 0.05; group: *F*(1,15)  =  0, *p*  =  1.0; group *x* context: *F*(4,60)  =  0.60, *p*  =  0.66).

Subjects' motivation (Figure 3C) displayed a strikingly different trend: objective good and bad groups showed no dissimilarity in ratings (two-way mixed-design ANOVA: context [repeated measure]: *F*(4,60)  =  29.4, *p* < 0.05; group: *F*(1,15)  =  0, *p*  =  1.0; group *x* context: *F*(4,60)  =  0.2, *p*  =  0.95). Yet context-dependant group ratings that were divided on the basis of subjective performance diverged significantly (two-way mixed-design ANOVA: context [repeated measure]: *F*(4,60)  =  38.7, *p* < 0.05; group: *F*(1,15)  =  0, *p*  =  1.0; group *x* context: *F*(4,60)  =  3.3, *p* <0.05). Subjective good subjects rated the high-gain contexts ($5/−$1, $5/−$5) equivalently, followed by the low-gain contexts, indicating their motivation rating solely depended on the gains. However, the subjectively bad group showed the reverse pattern, i.e. contexts involving high losses were more motivating than high-gain contexts, congruent with the notion that they believed themselves more likely to perform poorly. Also note that the ANOVA statistics indicate that grouping subjects by subjective as compared to objective performance—through the interaction of gain-loss context and performance group—accounts for a greater proportion of the variance in both preference and motivation ratings.

### Neuroimaging Findings

As this study chiefly concerns modulation of motor preparatory activity, the focus lies primarily upon sustained BOLD activity during the delay period that precedes the motor response. FMRI-responses elicited by the cue and the feedback stimulus are described in the supplemental results and discussion (Text S1).

### Motor Preparatory ROIs

**Figure 4:**
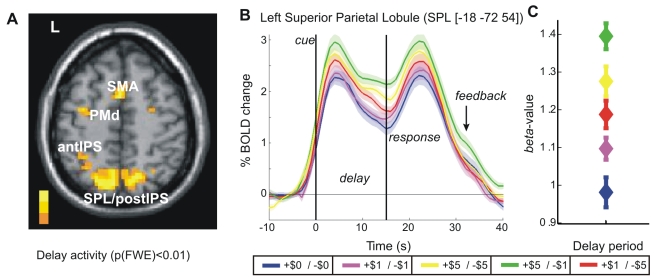
Modulation of delay activity by reward context. (A) Regions exhibiting significant delay period activity, across all gain-loss conditions (*p*(FWE) < 0.01, *k* > 5). (B) Average BOLD signal time-courses for different gain-loss contexts (shaded error bars: ±SE), extracted from left Superior Parietal Lobule (SPL) of each subject. Vertical lines demarcate delay period onset at 0 s and average movement onset at 15 s; combined “cues presentation” (gain-loss context cue, spatial cue, and mask) lasts 3.7 s prior to the onset of the delay period. (C) Average beta values (±SE) for the corresponding delay-period regressors, averaged across all subjects.

The primary analysis identified “*motor preparatory ROIs*” as those clusters that (i) exhibited increased levels of fMRI activity during the delay period (i.e. the time preceding a motor response), irrespective of the gain-loss context, and that (ii) have been previously shown to exhibit specific motor-preparatory activity (see Materials and Methods for details). By this approach, a group analysis revealed significant delay period activity in the left superior parietal lobule (SPL), along the medial bank and fundus of both the most posterior and most anterior aspects of the intraparietal sulcus (postIPS, antIPS), the dorsal premotor cortex (PMd), and the (pre-)supplementary motor area (SMA) (see Figure 4A and Table S1). While we later discuss that these areas exhibited delayed activity most likely due to their role in prospective motor planning, retrospective spatial memory and attention might have contributed to their activity as well.

Among the motor preparatory ROIs, the left SPL demonstrated the most robust delay period activity; left SPL BOLD time-courses averaged across all subjects (±SE), sorted by gain-loss context, are illustrated in Figure 4B. Expressed in %-signal change relative to the last 4 s of the initial fixation period, the exemplary time-courses of this region show four main components: (1) a transient (high-amplitude) signal increase time-locked to the cue, peaking approximately 6 s after cue-presentation; (2) a sustained level of activity during the delay period, but of a smaller magnitude than the earlier cue-related and the later movement-related peak amplitudes; (3) a transient (high-amplitude) signal increase time-locked to the initiation of movement, again peaking approximately 6 s after movement onset; and (4) a smaller transient increase time-locked to the feedback (receipt of reward/punishment), often obscured by the decay of the larger, movement-related signal.

To better isolate delay period modulations consequent of gain-loss contexts (without residual contributions from the cue epoch), the corresponding beta values are depicted in Figure 4C. As these beta values are regression coefficients that represent the “weight” of each predictor in order to best fit the observed signal relative to the residual baseline activity, they constitute a normalized estimate of the signal change due to each predictor—in this figure, the delay periods under each gain-loss context. Averaged over all subjects, the preferred high-gain/low-loss (+$5/−$1) context produced the largest signal. While this tentatively suggests that the BOLD response may reflect the value associated with the trial or subjects' preference rankings, the remaining gain-loss contexts do not generate levels of activity proportional to either the value model or subjective preference—most notably, the beta value associated with the low-gain/high-loss context (+$1/−$5) exceeds those associated with the low-gain/low-loss and neutral context (+$1/−$1 and +$0/−$0, respectively). This strongly suggests that the *absolute value* associated with successful trial completion may play an explicative role in shaping delay period responses.

**Figure 5:**
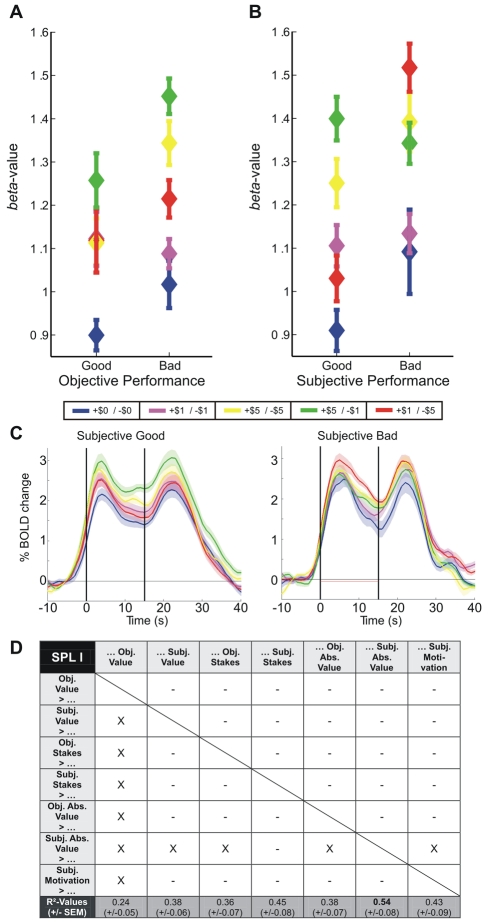
SPL activity correlates best with subjective absolute value. Left SPL delay period beta values for (A) objective good versus bad and (B) subjective good versus bad subjects. (C) Corresponding BOLD time-courses for subjective good (left) and bad (right) subjects (figure conventions as in Figure 4). (D) The chart depicts the average R^2^-values of the linear regression between the different explanatory models and individual subject's beta estimates in the left SPL for different reward contexts. Significant differences in R^2^-values of different models are indicated by “X.”

In a next step we tried to elucidate any relationship between objective and subjective performance levels and context-dependant delay period activity: the “objective good” group yielded no clear order of delay period beta values, except that the neutral context would have led to the lowest beta estimate in this and in all other groupings. The “objective bad” group exhibited a pattern similar to that of the overall group of subjects, with the high-gain/low-loss context (+$5/−$1) being highest, and the low-gain/high-loss context (+$1/−$5) being greater than the low-gain/low-loss and the neutral contexts (Figure 5A). This grouping on the basis of objective performance does not explain more of the overall variance in delay period beta values than when considering gain-loss contexts alone (two-way mixed-design ANOVA: context [repeated measure]: *F*(4,60)  =  4.595, *p* < 0.01; group: *F*(1,15)  =  0.475, *p*  =  0.5; group *x* context: *F*(4,60)  =  0.54, *p*  =  0.71).

Alternatively, subjects were divided according to their *subjective* performance estimate. Delay period beta values (Figure 5B) disclose a trend for an interaction between gain-loss context and subjective performance grouping (two-way mixed-design ANOVA: context [repeated measure]: *F*(4,60)  =  6.248, *p* < 0.001; group: *F*(1,15)  =  0.32, *p*  =  0.56; group *x* context: *F*(4,60)  =  2.267, *p*  =  0.07). For the “subjective good” group, the beta values of +$5 contexts exceed those of the +$1 contexts, with the highest beta associated with the +$5/−$1 context. For the “subjective bad” group, the −$5 contexts garner a larger hemodynamic response than the high-gain/low-loss context (+$5/−$1), which in turn produces a larger response than the low-gain/low-loss and the neutral context. Collectively considered, these findings concur best with the absolute value model for both subjective good and subjective bad performance (cf. Figure 2C). They do not concur with the value or stakes model, nor do they with the subjective rankings about preference and motivation. To demonstrate the task-dependent modulation of activity throughout the trial, Figure 5C renders the left SPL BOLD signal time-courses for both the subjective good and the subjective bad group.

**Figure 6:**
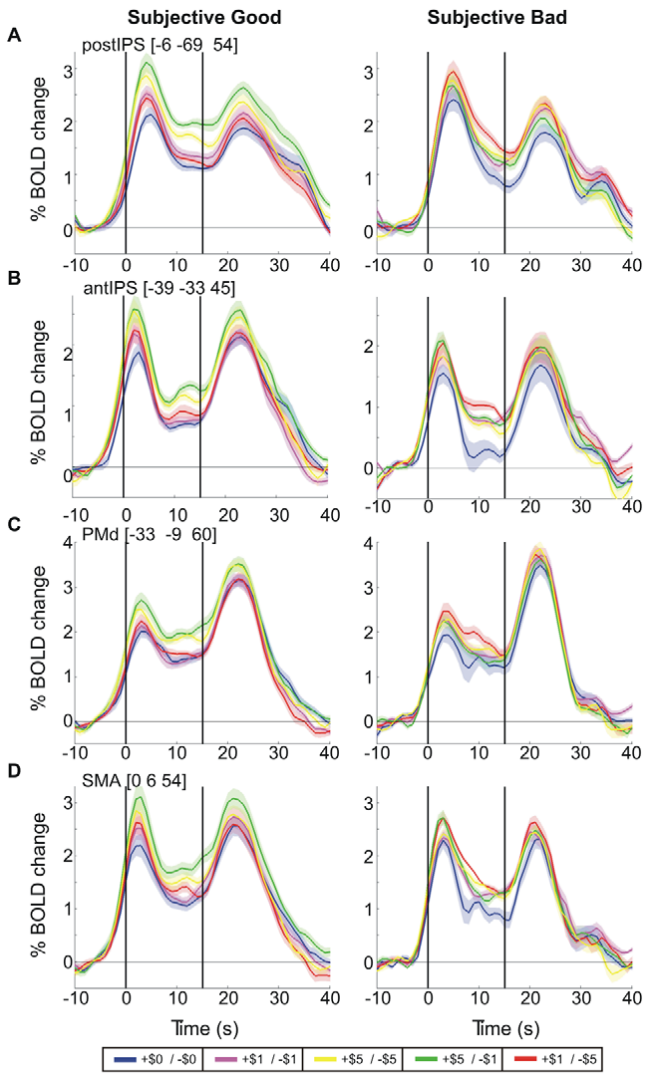
Reward modulation in motor preparatory ROIs depends on subjective performance estimates. BOLD time-courses for subjective good (left) and subjective bad subjects (right) are shown for motor preparatory ROIs: (A) Precuneus, (B) IPS, near its junction with POS, (C) PMd, and (D) SMA (figure conventions as in Figure 4).

The profile of BOLD activity in other motor preparatory ROIs echoed that in SPL. Figure 6 portrays the analogous time-courses, for subjective good and bad subjects, for left postIPS (Figure 6A), left antIPS (Figure 6B), left PMd (Figure 6C), and SMA (Figure 6D). Across these posterior parietal and premotor areas, neural activity developed similarly, likely reflecting a modulation of BOLD responses by the absolute value tied to task completion (compare Figure 2C). Amongst these areas, the left postIPS revealed the most robust context-dependent responses (Figure 6D). Moreover, supporting our findings for the left SPL, delay period beta values in neighboring left postIPS reveal a significant interaction between gain-loss context and subjective performance grouping (two-way mixed-design ANOVA: context [repeated measure]: *F*(4,60)  =  9.03, *p* < 0.001; group: *F*(1,15)  =  0.342, *p*  =  0.57; group *x* context: *F*(4,60)  =  2.589, *p* < 0.05) but not for objective performance grouping (two-way mixed-design ANOVA: context [repeated measure]: *F*(4,60)  =  6.597, *p* < 0.001; group: *F*(1,15)  =  2.119, *p*  =  0.17; group *x* context: *F*(4,60)  =  0.503, *p*  =  0.73). This implies that the absolute value model for subjective performance might account best for posterior parietal planning activity.

**Figure 7:**
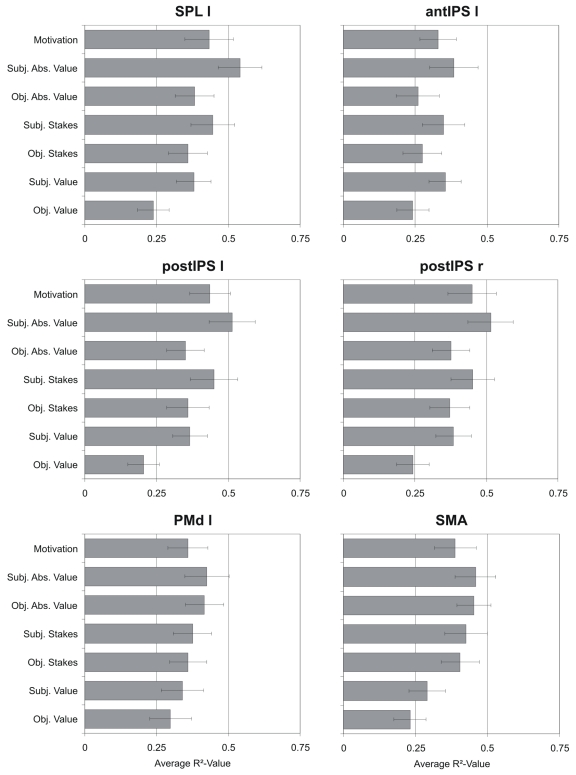
Subjective absolute value coding in motor preparatory ROIs. For each ROI the average R^2^-values of the linear regression between the different explanatory models and individual subject's beta estimates for different reward contexts are depicted.

To further corroborate these findings, a series of ROI analyses was conducted to directly probe context-dependent modulations: on a single-subject level the beta values for each reward context were extracted for a given ROI and entered into linear regression analyses. These analyses revealed individual coefficients of determination (R^2^-values) for several explanatory models that could account for the modulation of the delay-related beta estimates in a given ROI due to the gain-loss contexts. Separate models were calculated for modulations according to the value, the stakes, the absolute value, and the subjective motivation model. Since the earlier three models also incorporate estimates of performance, we calculated both “objective performance” and “subjective performance” models. For the “objective performance” models, these hypothesized modulations for each subject were determined by their objective performance and for “subjective performance” models, by their subjective performance estimate (see Experimental Procedures, Table 1 for values used for these hypothesized modulations). For all ROIs, subjects' beta estimates were best explained by the absolute value model which was based on subjective performance. This is evident from the average R^2^-values in Figure 7: in each ROI the respective R^2^-value for the subjective absolute value model was the highest. In other words, this model was the best to account for the variance of motor-preparatory activity due to our reward-context. For instance, it explained more than 50% of the variance in the left SPL and in the postIPS of both hemispheres. Conversely, the least amount of variance was captured by the objective value model. Finally, for all ROIs and for all performance-based models, those based on subjective performance estimates always explained more variance than their objective counterpart, i.e. the same model but based on objective-performance estimates.

**Table 1:** Rank assignment of gain-loss contexts for parametric modulation.

Gain-Loss Context	Value		Absolute Value		Stakes		Risk	Gains	Losses
Performance	Good	Bad	Good	Bad	Good	Bad			
+$5/−$1	5	5	5	3	4	3	3	3	2
+$5/−$5	4	2	4	4	5	5	4	3	3
+$1/−$5	1	1	3	5	3	4	3	2	3
+$1/−$1	3	3	2	2	2	2	2	2	2
+$0/−$0	2	4	1	1	1	1	1	1	1

These ranks were mean-corrected and then convolved with the canonical hemodynamic response function. Group random effects analyses disclosed voxels whose BOLD activation during the cue, delay, and response epochs significantly correlated with these parametric modulators, independent of a main effect for the respective task epoch.

In order to allow a statistical comparison between models, we performed a one-way repeated-measures ANOVA for each ROI, which was calculated across subjects' R^2^-values for the different models. In case of a significant influence of the factor “model,” additional pair-wise comparisons between models were performed (see Materials and Methods for details). A significant influence of the factor “model” was revealed for SPL (*F*(6,96)  =  2.7, *p* < 0.05), postIPS (left: *F*(6,96)  =  3.0, *p*<0.05; right: *F*(6,96)  =  2.41, *p* < 0.05), and the SMA (*F*(6,96)  =  2.6, *p* < 0.05). For the left SPL the results of the post-hoc comparisons between the different models are shown in Figure 5D. The figure reveals (i) that in the left SPL the subjective absolute value model explains significantly more variance than all other models but the subjective stakes model and (ii) that the objective expected value model performs significantly worse than all other models. The same principal pattern of results also surfaced for the postIPS in both hemispheres, except that the subjective absolute value model was not significantly better than the subjective motivation model (compare Table S2). All other ROIs display similar trends, though in these regions only a small subset of models could be statistically distinguished, if at all (i.e. for the SMA) (compare Figure 7 and Table S2).

Finally, we conducted a second set of full-brain group analyses to directly probe brain regions that exhibit context-dependent modulation. General linear models (GLMs) were defined for each individual subject that employed a single regressor for each task epoch. For the cue, delay, and response epochs, an additional regressor captured the hypothesized parametric modulation of the fMRI signal due to gain-loss contexts. Separate models were calculated for the value model, the stakes model, and the absolute value model (based both on subjective and objective performance estimates; see Table 1) as well as for subjects' preference and motivation. On the second level, group analyses exclusively utilized contrast images from individual subjects which assessed the beta values of each parametric regressor capturing the respective modulation of delay-related BOLD signals in accordance with each of our explanatory models. By this approach, all voxels in which a particular model could significantly account for delay period activity were mapped. Furthermore, we were able to directly contrast our main models using multiple pair-wise comparisons.

**Figure 8:**
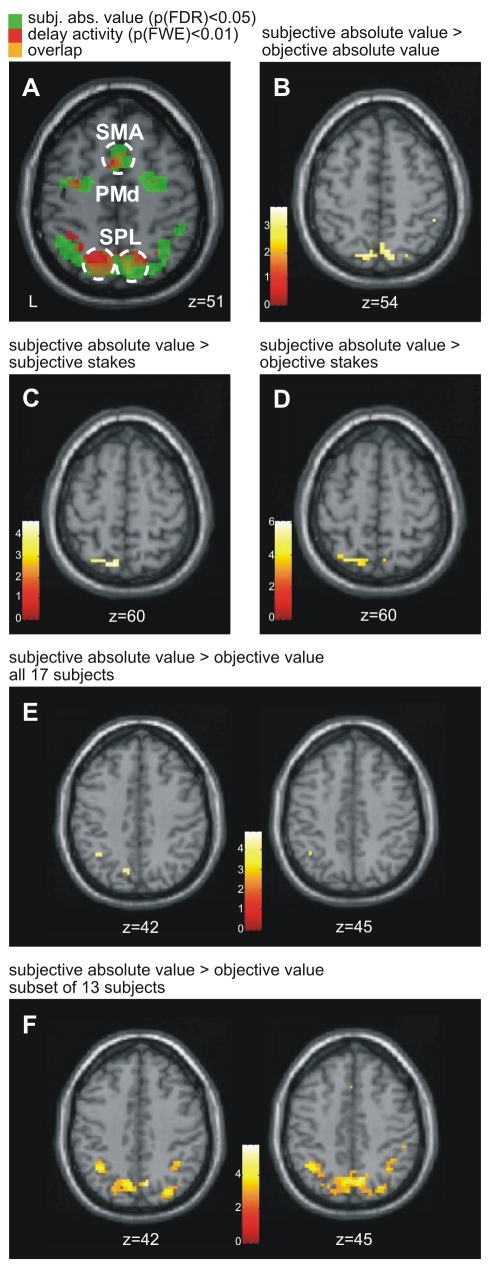
Subjective absolute value coding in Posterior Parietal Cortex. (A) Voxels revealing a significant main effect of the delay period are shown in red (*p*(FWE) < 0.01, *k* > 5); voxels revealing a significant parametric modulation of absolute value, based on subjective performance, are depicted in green (*p*(FDR) < 0.05, *k* > 5). Circled clusters of overlap (depicted in yellow) are significant at *p* < 0.05 (corrected at cluster level). (B–F) Cortical sites that exhibited significant differences when comparing between our six main models on the second level (*p*(FDR) < 0.05, *k* > 5; inclusive mask for delay period activity at *p*(FWE) < 0.01; *k* > 5 voxels [mask shown in red in A]). Note that the pairwise comparisons between models often suffered from the high degree of correlation between models in a subset of our subjects. Thus, the distinction between the models improved markedly by focusing on those subjects in whom the predictions of both models under comparison differed maximally. This principle is exemplified in (F): the comparison of subjective absolute value versus objective value, in which all subjects are included except those with both good subjective and good objective performance estimates, i.e. excluding those subjects in whom the predictions of both models converge (cf. Figure 2).

For second-level GLMs predicated upon stakes and value (either rooted in objective or subjective performance estimates) or predicated upon subjective preference and motivation, this contrast produced no significant voxels (up to an uncorrected voxel level threshold of *p*<0.05). However, confirming the results of our previous ROI analyses, absolute value models based on subjective performance yielded significant clusters in parietal and premotor cortex (*p*<0.05 corrected at cluster-level; k > 5 voxels; threshold at the voxel-level: *p*<0.05 FDR-corrected), rendered in green in Figure 8A (also compare Tables S1 and S3). Models of absolute value based on objective performance also highlighted a subset of these clusters, but these voxels did not survive the statistical threshold criteria. Superimposed on the statistical map for subjective absolute value in Figure 8A are the motor preparatory ROIs, which exhibited a significant main effect of the delay period (red). The extensive overlap suggests that these major motor preparatory ROIs were also the regions most significantly encoding subjective absolute value-related information.

To further assess the ability of one model to better account for the observed patterns of BOLD activation, paired *t*-test comparisons between our six main models (Figure 2) were performed for all possible model combinations. For example, in order to compare objective value and subjective value models, the two contrast images corresponding to the parametric modulation for the two models were extracted from each subject (first-level GLMs) and considered as pairs in the paired *t*-test comparison (resulting in 17 pairs for 17 subjects for each paired *t*-test). In this analysis, only the subjective absolute value model, when compared to other models, yielded significant activation: the contrasts of subjective absolute value > objective absolute value (Figure 8B), subjective absolute value > subjective stakes (Figure 8C), subjective absolute value > objective stakes (Figure 8D), and subjective absolute value > objective value (Figure 8E,F) all exhibited significant voxels within right and left SPL (*p*(FDR) < 0.05, inclusive mask for delay period activity at *p*(FWE) < 0.01; k > 5 voxels [Figure 8A]; also compare Table S4). No suprathreshold clusters for any other comparisons, including the inverse contrasts (e.g. objective value > subjective absolute value), were revealed.

## Discussion

To determine which aspects of an action's reward contingencies pertain to action preparation, human subjects were scanned while performing a difficult motor planning task with monetary consequences, contingent on task performance. Importantly, task demands were of sufficient complexity to generate a range of performance levels and robustly recruit motor preparatory areas, leading to several novel findings. We found that each subject performed at a consistent success level throughout the experiment. Yet performance across subjects differed significantly and—more interestingly—their subjectively perceived performance was not correlated with their actual performance. Moreover, subjective performance estimates better accounted for subjects' attitudes towards the gain-loss contingencies. Furthermore, our findings show differences in BOLD activity which are related to these gain-loss contingencies. As there was no evidence of any differences in behavioral responses between conditions, this BOLD modulation most likely reflected the subjects' evaluation of the predicted monetary consequences of their upcoming actions. Specifically, our imaging findings demonstrated that posterior parietal and premotor cortex assimilated the absolute value of a motor plan during the delay period. This absolute value was not predicated upon subjects' actual performance levels, but rather upon their subjective performance estimates.

### Involvement of Canonical Reward Structures in Encoding Reward Context

Areas in striatum and orbitofrontal cortex exhibited significant responses to the cue and outcome epochs of the task but showed no sustained delay period activity. Prior imaging studies investigating these regions did not generally employ delay periods long enough to unambiguously disentangle neural signals generated in these different task epochs. In contrast, here the profile of orbitofrontal and striatal BOLD activity did correspond to the time-resolved character of single-unit investigations [39]–[42]: regions in the striatum and orbitofrontal cortex revealed similar reward-contingent modulation. Namely, in most regions the cue response amplitude reflected the objective value predicted by the context cue (supplemental results in Text S1, Table S5 and Figure S1; also compare Figure S2). Moreover, both the striatum and orbitofrontal cortex differentiated between rewarding and punishing outcomes and their relative size (Table S6 and Figure S3), consonant with their purported role in utilizing feedback in the control of motivated behavior [9],[10]. In summary, these findings contribute to the idea that orbitofrontal and striatal areas may process information highly relevant for guiding goal-directed action but do not necessarily participate in planning and preparing movements per se.

### Role of Motor Preparatory Regions in Encoding Action Outcomes

In the current investigation, we identified ROIs with sustained BOLD-activity in the delay period as the putative neural substrates of motor preparation. Our approach mimicked the design of a related study, which demonstrated that the delay-activity in corresponding regions can be distinguished from activity related to both preceding visual events and subsequent motor responses. Moreover, in the same study we were able to demonstrate that BOLD-activity in the respective ROIs could not be explicated solely by concurrent processes such as visuospatial attention or working memory. These regions—in particular the left SPL—showed greatest activity in conditions requiring that spatial cues be encoded with respect to a motor plan [43]. Here we asked whether the same regions would also display a modulation of delay period activity due to gain-loss consequences.

Our results revealed that, throughout the motor-preparatory areas, BOLD signal amplitudes for trials in which actions could either endow high gains or high losses surpassed those in more neutral trials. Moreover, the high-gain/low-loss (highest valued) context elicited the most activity in subjects who believed themselves more likely to succeed, whereas the low-gain/high-loss (lowest valued) context produced the greatest BOLD response in subjects who believed themselves more likely to fail. Across ROIs this BOLD activity pattern was always best explained by the subjective absolute value model and worst by the objective value model.

### Action Planning in Posterior Parietal Cortex

Clusters throughout SPL exhibited the most robust delay period activity, consistent with our previous work, and also best exemplified context-dependent modulation of this activity. These clusters closely correspond to areas in PPC localized as a putative human analog of the macaque parietal reach region (PRR) [Glidden, H.K., Rizzuto, D.S. and Andersen, R.A., 2009, in revision]. Interestingly, recordings in the macaque have shown that, before the monkey performs a reach, PRR demonstrates activity related to expected value of the outcome [33]. These findings speak to a general role of PPC in encoding expected outcomes of actions as a facet of action plans. Accordingly, a plethora of monkey electrophysiology studies have examined how expected reward influences neural activity in PPC, with a particular focus on visuo-oculomotor behavior. Firing rates in LIP, the region of PCC thought particularly devoted to the representation of eye movements [27], reflect behaviorally relevant information in saccadic paradigms probing target detection, expected value, relative utility, and internal choices [30],[44],[45]. In demonstrating and characterizing outcome-related modulation in *human* PPC, our data augment these previous findings, and significantly extend their interpretation by considering PPC responses to both penalties and rewards, as well as their dependency on objective and subjective performance estimates.

### Premotor Cortex Activity in Delayed-Response Tasks

Another region traditionally implicated in various aspects of action planning and preparation is the PMd. In the macaque premotor area, the question of value versus motivation encoding has been investigated through single-cell recordings [35]. In this decision-making study, monkeys made saccades to indicate their choice between targets yielding either a punishment (a time-out) or a fluid reward. Neurons in premotor cortex fired robustly in anticipation of both large rewards and punishments, a finding deemed reflective of “motivation.” The predictions of motivation as put forth by those authors would coincide with those of absolute value and stakes/risk as defined in our experimental framework. Given this correspondence, human dorsal premotor areas (PMd, SMA) manifested the same trend.

### The Influence of Subjective Biases on Motor Preparatory Activity

An added dimension in the exposition of motor preparatory activity stems from the impact exerted by the subjects' perceived performance. Since we did not expect a major impact of this factor, the collected self-estimate of subjects' perceived performance (“good” versus “bad”) was rather rudimentary. Nevertheless, we were able to demonstrate a significant explanatory influence of these subjective performance estimates on the preparatory BOLD activity. Specifically, in this study, the subjects' “conception of acts, outcomes and contingencies” [46] deviated from the objective likelihood of outcomes. The perceived (subjective) performance was of even greater importance than the actual (objective) performance in explicating both subjective attitudes and neural data, attesting to a strong framing effect. That attitudes and beliefs about the likelihood of outcomes affect behavior or decision-making is not surprising. Psychologists have long posited that humans exploit certain heuristics or simplifying beliefs under conditions when available information is incomplete or overly complex [47]. However, in our experimental scenario, variability in outcomes stems from subjects' abilities and all information necessary to track performance is provided. Nonetheless, our results suggest that motor-preparatory regions seem more susceptible to subjective beliefs than to objective performance.

### Possible Limitations

One limitation of our study stems from the equivocal interpretation of the BOLD signal as a marker for neuronal activity: positive BOLD responses may derive from neuronal excitation, neuronal inhibition, or other factors. Thus, actual neuronal activity could conceivably resemble a (signed) value model (with stronger excitation for higher gains and stronger suppression for larger punishments), whereas the BOLD signal would reflect an absolute value model, with larger responses for both stronger excitation and inhibition. Such a contention broadly constrains all fMRI studies considering BOLD activation as a function of incentive salience. Two lines of reasoning, however, controvert the aforementioned interpretation: First, given the design of the present study, the task demands render a major contribution of neuronal inhibition to the delay-related BOLD signals less likely—specifically, an accurate movement was identically requisite both to achieve gains and to avoid punishments. Thus our subjects were strongly motivated to prepare (but not to inhibit) a movement whenever the possibility of either a large gain or a large loss existed—mirroring the absolute value model. Second, the delay-related BOLD signals clearly resemble electrophysiological findings in monkey PMd [35], which also showed excitatory preparatory activity related to subjects' motivation and thus consonant with the absolute value rather than the value model (see above). Of course, additional electrophysiological evidence is needed to indisputably substantiate the notion of an absolute value coding in PPC. Furthermore, additional experiments are needed to allow a more clear-cut separation between the subjective absolute value model and alternative models: it could not be statistically distinguished from the subjective stakes model in any of our ROIs. Similarly, the second-level model comparison revealed significant differences between the subjective absolute value model and all other models but the subjective value model. Successful statistical distinction was particularly difficult because our models were highly correlated (cf. Figure 2; Figure 8E,F) and some even converged in subsets of subjects: in those subjects with the same objective and subjective performance estimates (*N*  =  8), subjective and objective model variants would yield the same predictions. Nevertheless, the fact that the subjective absolute value model always explained a higher amount of variance in all ROIs under investigation clearly underlines the import of this model.

Another limitation refers to the separation of the various cognitive processes that potentially underlie the delay-related BOLD activity. While we have concurrently provided evidence for a major role of our ROIs in motor planning (see above), action preparation within the current paradigm also required attention and spatial working memory—processes which might contribute to delay-related BOLD activity as well [48]–[50]. Thus, this study ultimately cannot disambiguate the preparatory processes that are modulated by the reward context. Yet this modulation of preparatory activity is still in accordance with the formulation of a specifically defined quantity, namely subjective absolute value.

### Implications for Response Selection

Collectively taken, lesion, electrophysiology, and imaging studies highlight the function of PPC in assimilating relevant, non-sensory information with sensory- and movement-specific representations, asserting its role in decision making related to action. Moreover, recent studies advocate PPC's capacity to simultaneously encode competing motor plans [51], while incorporating signed value information into these alternative action representations [30]–[32],[52],[53]. Thus PPC seems well situated to render decisions between behavioral alternatives. Yet the apparent encoding of subjective absolute value instead of objective value in our task suggests that PPC represents behavioral plans that flexibly integrate the different types of information provided by other decision-related areas (e.g. signed value information represented in the striatum and orbitofrontal cortex or absolute value information; cf. [7]) in a context-specific manner. In the context of our task (when only a single but rather demanding response was required), the observed modulation of PPC by subjective absolute value might facilitate behavior by mobilizing resources for those prospective actions that are most optimal—either for pursuing gains or for avoiding losses. Thereby, expectations about behavioral outcomes, derived from generalizations of precedent predictive relationships, were especially relevant for investing in a current course of action. Subjective cognitive biases can distort these expectations or generalizations; if they also distort activity in those regions encoding action representations, they contribute to motor preparation and, presumably, to response selection. In this manner, the observed import of subjective performance estimates, which here were not correlated with objective performance measures, may form one of the ways in which people seem to deviate from rationality in their goal-directed behaviors [54],[55], taking actions that appear contrary to logic or self-interest.

## Materials and Methods

### Subjects

Seventeen subjects (7 males, 10 females), ranging from 17–27 years old, participated in the experiment. All subjects were right-handed and exhibited normal or corrected-to-normal visual acuity. Subjects received a $15 recompense for completing all training and scanning, in addition to their earnings during the experiment.

### Ethics Statement

Participants provided informed consent in accordance with the Caltech Institutional Review Board guidelines.

### Experimental Setup and Behavioral Control

All visual stimuli were back-projected onto a translucent screen (22 deg×16 deg visual angle) by using a video projector (800×600 pixels, 60 Hz). Subjects viewed the visual stimuli via a mirror that was mounted on the head coil of the MRI scanner (viewing distance 1,150 mm). Stimuli were generated on a windows PC using “Cogent Graphics” developed by John Romaya at the LON at the Wellcome Department of Imaging Neuroscience. Subjects positioned a fiber optic trackball (Current Designs, Pennsylvania) upon their torso, holding the device in place with their right hand and adjusting the exact placement for comfort. All subjects used their right index finger to make “finger reaches,” manipulating the trackball to move the cursor on the screen. These trackball movements were recorded and analyzed online in MATLAB.

The experimental task required subjects to dissociate arm and eye movements, demanding central visual fixation throughout each trial (subjects were allowed to make eye movements during the intertrial interval). A miniature infrared eye camera (60 Hz sampling rate; Resonance Technologies, California) placed inside the headcoil monitored eye movements during all scanning sessions. Recorded eye behavior (ViewPoint, Arrington Research, Arizona) was then analyzed offline in MATLAB.

### Experimental Task

Figure 1 depicts the task structure and timing. Each trial began with an initial fixation period (15 s on average, randomly jittered between 14 and 16 s). The gain and loss context for the current trial was then presented above and below the fixation point, respectively, for 1.5 s. Next, the spatial cues, 9 squares, radially equidistant from the fixation spot, were presented (1.2 s). To prevent subjects from memorizing a set number of locations, two configurations of squares, rotated 20 degrees with respect to one another, were randomly interleaved across trials. Of these 9 squares, 5 were “hollow” (containing an inner black square), denoting them as targets for the upcoming finger reaches. In addition, these 5 square targets varied in size, specifying the order (from smallest to largest) in which the subjects should move towards them. A visual mask (80 randomly placed white squares) displayed for 1 s erased any iconic visual memory of the targets. The ensuing delay period, during which subjects were reminded of the gains and losses for the trial, lasted 15 s on average, again jittered between 14 s and 16 s, complementary to the baseline fixation duration, to ensure all trials were of equal length. Ultimately, the response screen appeared, serving as the “go” signal, with 9 identical squares in the same locations as the squares during presentation of the spatial cues. A circular cursor was also shown, centered on the fixation point. At this time, subjects moved the cursor in a center-out fashion (from center to target, back to center, to next target, etc.) sequentially to the 5 targets, in the order previously instructed. Subjects were allowed 10 s in which to complete the task. Finally, subjects received feedback: the gain amount if they successfully acquired all targets; the loss amount otherwise.

This experiment utilized five gain-loss contexts: +$0/−$0, +$1/−$1, +$1/−$5, +$5/−$1, and +$5/−$5. Each gain-loss context trial type occurred 6 times per run, producing a total of 30 trials per run; the order of trial types was pseudorandomized and counterbalanced. Subjects trained extensively, performing 5 practice runs outside the scanner and 1 practice run within the scanner. They then completed 2 runs during scanning. To promote constant performance throughout the task, subjects were additionally instructed to “do their best” on all trials, irrespective of the gain-loss context. Given this instruction and exhaustive practice, individual subjects' performance on the task during scanning remained stable (see Results). Each subject's mean performance is therefore taken as her/his fixed probability of success.

Immediately after the scanning session and before ascertaining any information about their actual performance or net winnings, subjects answered a questionnaire: (1) whether they paid attention to the gain-loss contexts and (2) whether they had performed well on the task, and net made money; performed poorly, and net lost money; or roughly broke even (∼50% performance; note that this option was never chosen). They also ranked the 5 gain-loss contexts with respect to preference (under which context trial types they preferred working, from most [5] to least [1]) and motivation (under which context trial types they wanted to perform well, from most [5] to least [1]).

### Functional and Anatomical Imaging

Echo-planar functional images were acquired in a Siemens 3-Tesla Trio scanner at Caltech's Brain Imaging Center, using an 8-channel head coil. The scan volume provided full coverage of cortical and subcortical structures in 32 axial slices, except that it did not cover the cerebellum in its entirety (slice thickness  =  3.5 mm, gap  =  0 mm, in-plane voxel size  =  3×3 mm, TR  =  2,000 ms, TE  =  30 ms, flip angle  =  90°, FOV  =  192×192, resolution  =  64×64). Subjects completed 2 runs, each 1,487 s in duration. Anatomical images were acquired using a T1-weighted MP-RAGE sequence with the same head coil used for functional image collection. The whole brain volume was scanned in 176 slices (slice thickness  =  1 mm, gap  =  0 mm, in-plane voxel size  =  1×1 mm, TR  =  1,500 ms, TE  =  3.05 ms, FOV  =  256×256, resolution  =  256×256).

### Data Preprocessing and Analysis

Functional data preprocessing, conducted through SPM5 (Wellcome Department of Imaging Neuroscience, Institute of Neurology, London, UK), included slice scan time correction, 3D motion correction, and linear trend removal. Mean EPI images were coregistered to whole-brain high resolution T1-weighted structural images (1×1×1 mm) acquired for all subjects. Anatomical images were spatially normalized to a standard T1 template; the same normalization parameters were then applied to all functional images. All EPIs received additional intensity normalization, spatial smoothing (7 mm Gaussian kernel), and temporal high pass filtering (0.0078 Hz).

After data preprocessing, two types of across-subjects analyses were performed: (1) an “ROI-based” approach, delimiting brain regions of interest for each epoch of the task, allowing characterization of BOLD modulation due to gain-loss contexts in those regions, and (2) a “whole-brain” approach, exposing all regions that displayed a predicted modulation due to gain-loss contexts.

(1) The ROI-based approach defined motor preparatory areas on the basis of the linear combination of all delay period covariates, i.e. under all gain-loss contexts. Areas involved in cue processing were delineated accordingly (significant positive beta value for all cue predictors). The within-subject “ROI” localizer utilized a GLM (Friston et al. 1995) incorporating 21 total predictors of interest: the cue period for each gain-loss context (5 cue predictors), delay period for each gain-loss context (5 delay predictors), response period for each gain-loss context (5 response predictors), and outcome period for each magnitude of reward or punishment (5 outcome predictors: +$5, −$5, +$1, −$1, $0). These boxcar predictors were convolved with the SPM 5 canonical hemodynamic response function. Statistical detection of BOLD activation related to different task epochs (cue, delay, and response) was based on a across-subjects, random effects model with a statistical threshold at *p(FWE-corrected) *<0.01 (k > 5). In order to determine the relevant regions of interest (ROIs) that exhibit motor preparatory BOLD-activity in the delay period, we only selected regions that (i) were also significant on the clusters level (*p*<0.05, corrected) and that (ii) have been shown to exhibit motor preparatory activity in an earlier study of comparable design [43]. A full list of those areas that survived criterion (i) is provided in Table S1. Those areas of the table that were previously characterized as brain regions contributing to motor preparation (criterion (ii)) are in italics. Our method thus conservatively highlighted regions manifesting a consistent deviation from baseline during the delay period without biases imposed by any predetermined hypothesis as to the modulation expected during this task epoch.

In a next step, in each individual and for each of these functionally defined ROIs the mean beta weights of the various delay-period regressors and the BOLD signal time-courses were extracted for a 3 mm radius sphere around the voxel exhibiting a local maximum *t*-value for the main delay-activity contrast (both normalized to %-signal change). In addition, we used the individual beta values to perform a series of linear regression analyses. For each ROI and for each subject we calculated coefficients of determination (R^2^-values) for several explanatory models that could account for the modulation of the delay-related beta estimates in a given ROI due to the gain-loss contexts, namely the objective value, subjective value, objective stakes, subjective stakes, objective absolute value, subjective absolute value, and motivation model. In order to compare our explanatory models, we first normalized subjects' R^2^-values in order to eliminate any between-subjects variance in our within-subject design. Specifically, normalization was based on the deviation between a subject's (i) overall mean (Mi), computed across that subject's R^2^-value for each model, and the grand mean (GM) for the entire sample of subjects. This deviation was subtracted from the subject's R^2^-value in each condition (j): R^2^ij – (Mi – GM). Afterwards we performed a one-way ANOVA on the normalized R^2^-values and adjusted the degrees of freedom in a way that accounts for the within-subject design. Note that this procedure is formally equivalent to a one-way repeated measures ANOVA. In case of a significant influence of the factor “model,” additional pair-wise comparisons between models were performed (one-tailed tukey-kramer tests, significance threshold: *p* < 0.05 corrected).

(2) The second type of hypothesis-driven whole-brain analyses were predicated upon explicit suppositions as to BOLD signal modulation. Each respective analysis encompassed a distinct within-subject GLM for each relevant reward-related model (e.g. value, stakes, absolute value, etc.). These GLMs employed only four main predictors, one for each epoch of the task: cue, delay, response, and outcome. Additional predictor(s), for each of the first three epochs, modeled the hypothetical reward-related, parametric modulation(s) due to each trial's gain-loss context. For the last trial-epoch (i.e. the outcome epoch), additional predictors also captured any modulation due to the magnitude and the valence of feedback. Table 1 summarizes the respective parameters (rank numbers from low to high) for the different gain-loss contexts. For the value model, the stakes model, and the absolute value model, different parameters for good and bad performance were utilized; these parameters were drawn from averaging over 50%–100% performance (good) and 0%–50% performance levels (bad), respectively (compare Figure 1). Thus, in “objective performance” models, subjects were assigned “good” parametric modulation orders if they net won money, and “bad” otherwise. Similarly, in “subjective performance” models, subjects were assigned “good” parametric modulation orders if they believed they had net won money, and “bad” otherwise. For objective performance, an additional GLM, using ranks determined by each subject's actual performance, was analyzed. However, since subjective estimates can most conservatively be grouped as “above 50%” or “below 50%,” all results reported here use this binary grouping for both objective and subjective performance models, permitting better comparison between subjects' actual (objective) and perceived (subjective) performances. Models for “gains only” and “losses only” were also conducted to capture valence-selective modulations, addressing the possibility that separate systems respond to reward and punishment, respectively. Finally, we calculated models that included subjects' motivation ratings, subjects' preference ratings, and pair-wise interactions of the two, to see if they better accounted for the observed BOLD activity. All ranks were mean-corrected. Only those models significantly accounting for BOLD activation patterns across subjects (i.e. on the second-level) are discussed.

To account for observed BOLD modulations that may be ascribed to behavior, performance-related regressors were included in all second-level models, capturing (1) success (1 for successful trial completion, 0 otherwise), (2) reaction time latencies for each trial, and (3) total time required for motor response on each trial.

To further assess the ability of one model to better account for the observed patterns of BOLD activation, paired *t*-test comparisons between our six main models (Figure 2) were performed for all possible combinations. Specifically, different GLMs were estimated for each subject and for each model of interest as explained in detail above. From each GLM, the contrast image of interest (i.e., the image capturing the amount of parametric modulation of the delay-related fMRI activity as predicted by the particular model) was extracted for each subject and entered in the respective paired *t*-test analyses calculated across subjects (i.e. on the second level). For example, in order to compare objective value and subjective value models, the two contrast images corresponding to the two models were extracted from each subject (first-level GLMs) and considered pairs in a second-level paired *t*-test. All possible combinations of models were assessed; again, only those models producing significant differences are reported.

## Supporting Information

Figure S1 **BOLD time-courses of the left dorsal striatum for** ***objective*** **good and bad subjects.** The time-course over the entire trial duration is presented on top; two corresponding graphs that zoom in on the cue-related response (0 s denoting onset of gain-loss context cue, black lines) are depicted below. For the respective time-courses according to the *subjective* performance grouping, please refer to Figure S2.
(0.08 MB PDF)

Figure S2 **Dorsal striatal BOLD signal time-courses for** ***subjective*** **good and bad subjects.** The time-course over the entire trial duration is presented, with black lines indicating the onset of gain-loss context cue presentation. Note that unlike in Figure S1, which was divided on the basis of the *objective* performance, subjective grouping led to a larger overall variance (error bars).
(0.05 MB PDF)

Figure S3 **Orbitofrontal cortex BOLD signal time-courses.** The upper panel depicts time-courses for the entire trial duration; below a graph that zooms in on the feedback-related response is shown. Black lines at time 0 s correspond to the onset of the feedback information.
(0.04 MB PDF)

Table S1 **Brain regions exhibiting a significant contribution to the delay period.** All areas that were considered in our ROI analysis are in *italics* (*p* < 0.05 corrected at cluster level; *k* > 5 voxels; threshold at voxel-level: *p* < 0.01 FWE-corrected).
(0.01 MB PDF)

Table S2 **Average R^2^-values of the linear regression between explanatory models and individual subject's beta estimates for different reward contexts.** Significant differences in R^2^-values of different models are indicated by “X.” Separate tables are provided for all ROIs that showed a significant difference in R^2^-values across models, namely the left and right posterior IPS (pIPS l & pIPS r) and the SMA. The table for SPL is provided in the main manuscript (Figure 5D).
(0.03 MB PDF)

Table S3 **Parametric modulation of the delay-related BOLD-signal.** Only regions that exhibit a significant (*p* < 0.05 corrected at cluster level; *k* > 5 voxels; threshold at voxel-level: *p* < 0.05 FDR-corrected) correlation with our parametric modulators are listed.
(0.01 MB PDF)

Table S4 **Regions that exhibited a significant difference between models.** Second-level analyses were based on individual subject GLMs including a single explanatory model (*p* < 0.05 corrected at cluster level *p* < 0.05; *k* > 5 voxels; threshold at voxel-level: *p* < 0.05 FDR-corrected; inclusive mask for delay period activity at *p* < 0.01 FWE-corrected; *k* > 5 voxels).
(0.02 MB PDF)

Table S5 **Parametric modulation of the cue-related BOLD-signal.** Only regions that exhibit a significant (*p* < 0.05 corrected at cluster level; *k* > 5 voxels; threshold at voxel-level: *p* < 0.05 FDR-corrected) correlation with our parametric modulators are listed.
(0.01 MB PDF)

Table S6 **Parametric modulation of the outcome-related BOLD-signal.** Only regions that exhibit a significant (*p* < 0.05 corrected at cluster level; *k* > 5 voxels; threshold at voxel-level: *p* < 0.001 uncorrected) correlation with our parametric modulators are listed.
(0.01 MB PDF)

Text S1 **Supplemental results and discussion.** We provide additional results about fMRI responses elicited by the contextual gain-loss cues and also describe fMRI responses to the reward outcome. Finally, we discuss the involvement of canonical reward structures in the encoding of the reward context.
(0.06 MB DOC)

## Abbreviations

antIPS: anterior aspect of the intraparietal sulcus
BOLD: blood oxygenation level dependent
fMRI: functional magnetic resonance imaging
GLM: general linear model
IPS: intraparietal sulcus
LIP: lateral intraparietal area
PMd: dorsal premotor cortex
postIPS: posterior aspect of the intraparietal sulcus
PPC: posterior parietal cortex
ROI: region of interest
SMA: supplementary motor area
SPL: superior parietal lobule

## References

[1] Daw N. D, O'Doherty J. P, Dayan P, Seymour B, Dolan R. J (2006). Cortical substrates for exploratory decisions in humans.. Nature.

[2] Ernst M, Nelson E. E, McClure E. B, Monk C. S, Munson S (2004). Choice selection and reward anticipation: an fMRI study.. Neuropsychologia.

[3] Kable J. W, Glimcher P. W (2007). The neural correlates of subjective value during intertemporal choice.. Nat Neurosci.

[4] Hampton A. N, Adolphs R, Tyszka M. J, O'Doherty J. P (2007). Contributions of the amygdala to reward expectancy and choice signals in human prefrontal cortex.. Neuron.

[5] Knutson B, Westdorp A, Kaiser E, Hommer D (2000). FMRI visualization of brain activity during a monetary absolute value delay task.. Neuroimage.

[6] Knutson B, Adams C. M, Fong G. W, Hommer D (2001). Anticipation of increasing monetary reward selectively recruits nucleus accumbens.. J Neurosci.

[7] Cooper J. C, Knutson B (2008). Valence and salience contribute to nucleus accumbens activation.. Neuroimage.

[8] Jensen J, McIntosh A. R, Crawley A. P, Mikulis D. J, Remington G, Kapur S (2003). Direct activation of the ventral striatum in anticipation of aversive stimuli.. Neuron.

[9] Delgado M. R, Nystrom L. E, Fissell C, Noll D. C, Fiez J. A (2000). Tracking the hemodynamic responses to reward and punishment in the striatum.. J Neurophysiol.

[10] O'Doherty J, Kringelbach M. L, Rolls E. T, Hornak J, Andrews C (2001). Abstract reward and punishment representations in the human orbitofrontal cortex.. Nat Neurosci.

[11] O'Doherty J. P, Dayan P, Friston K, Critchley H, Dolan R. J (2003). Temporal difference models and reward-related learning in the human brain.. Neuron.

[12] Delgado M. R, Locke H. M, Stenger V. A, Fiez J. A (2003). Dorsal striatum responses to reward and punishment: effects of valence and magnitude manipulations.. Cogn Affect Behav Neurosci.

[13] Delgado M. R, Stenger V. A, Fiez J. A (2004). Motivation-dependent responses in the human caudate nucleus.. Cereb Cortex.

[14] Rolls E. T, McCabe C, Redoute J (2008). Expected value, reward outcome, and temporal difference error representations in a probabilistic decision task.. Cereb Cortex.

[15] Berns G. S, McClure M. S, Pagnori G, Montague P. R (2001). Predictability modulates human brain response to reward.. J Neurosci.

[16] McClure S. M, Berns G. S, Montague P. R (2003). Temporal prediction errors in a passive learning task activate human striatum.. Neuron.

[17] D'Ardenne K, McClure S. M, Nystrom L. E, Cohen J. D (2008). BOLD responses reflecting dopaminergic signals in the human ventral segmental area.. Science.

[18] Jensen J, Smith A. J, Willeit M, Crawley A. P, Mikulis D. J (2007). Separate brain regions code for salience vs. valence during reward prediction in humans.. Hum Brain Mapp.

[19] O'Doherty J. P, Buchanan T. W, Seymour B, Dolan R. J (2006). Predictive neural coding of reward preference involves dissociable responses in human ventral midbrain and ventral striatum.. Neuron.

[20] Bray S, O'Doherty J (2007). Neural coding of reward-prediction error signals during classical conditioning with attractive faces.. J Neurophysiol.

[21] Breiter H. C, Aharon I, Kahneman D, Dale A, Shizgal P (2001). Functional imaging of neural responses to expectancy and experience of monetary gains and losses.. Neuron.

[22] Ramnani N, Miall R. C (2003). Instructed delay activity in the human prefrontal cortex is modulated by monetary reward expectation.. Cereb Cortex.

[23] Tricomi E. M, Delgado M. R, Fiez J. A (2004). Modulation of caudate activity by action contingency.. Neuron.

[24] Zink C. F, Pagnoni G, Martin-Skurski M. E, Chappelow J. C, Berns G. S (2004). Human striatal responses to monetary reward depend on saliency.. Neuron.

[25] O'Doherty J, Dayan P, Schultz J, Deichmann R, Friston K (2004). Dissociable roles of ventral and dorsal striatum in instrumental conditioning.. Science.

[26] Bjork J. M, Hommer D. W (2007). Anticipating instrumentally obtained and passively-received rewards: a factorial fMRI investigation.. Behav Brain Res.

[27] Andersen R. A, Cui H (2009). Intention, action planning, and decision making in parietal-frontal circuits.. Neuron.

[28] Shadlen M. N, Newsome W. T (2001). Neural basis of a perceptual decision in the parietal cortex (area LIP) of the rhesus monkey.. J Neurophysiol.

[29] Gold J. I, Shadlen M. N (2001). Neural computations that underlie decisions about sensory stimuli.. Trends Cogn Sci.

[30] Platt M. L, Glimcher P. W (1999). Neural correlates of decision variables in parietal cortex.. Nature.

[31] Sugrue L. P, Corrado G. S, Newsome W. T (2004). Matching behavior and the representation of value in the parietal cortex.. Science.

[32] Dorris M. C, Glimcher P. W (2004). Activity in posterior parietal cortex is correlated with the relative subjective desirability of action.. Neuron.

[33] Musallam S, Corneil B. D, Greger B, Scherberger H, Andersen R. A (2004). Cognitive control signals for neural prosthetics.. Science.

[34] Andersen R. A, Hwang E. J, Mulliken G. H (2010). Cognitive neural prosthetics.. Ann Rev Psychology.

[35] Roesch M. R, Olson C. R (2004). Neuronal activity related to reward value and motivation in primate frontal cortex.. Science.

[36] Wrase J, Kahnt T, Schlagenhauf F, Beck A, Cohen M. X (2007). Different neural systems adjust motor behavior in response to reward and punishment.. Neuroimage.

[37] Samanez-Larkin G. R, Gibbs S. E, Khanna K, Nielsen L, Carstensen L. L (2007). Anticipation of monetary gain but not loss in healthy older adults.. Nature Neurosci.

[38] Trommershäuser J, Maloney L. T, Landy M. S (2008). Decision making, movement planning and statistical decision theory.. Trends Cogn Sci.

[39] Schultz W, Tremblay L, Hollerman J. R (2000). Reward processing in primate orbitofrontal cortex and basal ganglia.. Cereb Cortex.

[40] Kobayashi S, Kawagoe R, Takikawa Y, Koizumi M, Sakagami M, Hikosaka O (2007). Functional differences between macaque prefrontal cortex and caudate nucleus during eye movements with and without reward.. Exp Brain Res.

[41] Samejima K, Ueda Y, Doya K, Kimura M (2005). Representation of action-specific reward values in the striatum.. Science.

[42] Hikosaka O, Nakamura K, Nakahara H (2006). Basal ganglia orient eyes to reward.. J Neurophysiol.

[43] Lindner A, Iyer A, Kagan I, Andersen R. A (2010). Human posterior parietal cortex plans where to reach and what to avoid.. J Neurosci.

[44] Shadlen M. N, Newsome W. T (1996). Motion perception: seeing and deciding.. Proc Natl Acad Sci U S A.

[45] Coe B, Tomihara K, Matsuzawa M, Hikosaka O (2002). Visual and anticipatory bias in three cortical eye fields of the monkey during an adaptive decision-making task.. J Neurosci.

[46] Tversky A, Kahneman D (1981). The framing of decisions and psychology of choice.. Science.

[47] Tversky A, Kahneman D (1974). Judgement under uncertainty: heuristics and biases.. Science.

[48] Maunsell J. H (2004). Neuronal representations of cognitive state: reward or attention?. Trends Cogn Sci.

[49] Bendiksby M. S, Platt M. L (2006). Neural correlates of reward and attention in macaque area LIP.. Neuropsychologia.

[50] Curtis C. E (2006). Prefrontal and parietal contributions to spatial working memory.. Neuroscience.

[51] Cui H, Andersen R. A (2007). Posterior parietal cortex encodes autonomously selected motor plans.. Neuron.

[52] Gershman S. J, Pesaran B, Daw N. D (2009). Human reinforcement learning subdivides structured action spaces by learning effector-specific values.. J Neurosci.

[53] Serences J. T (2008). Value-based modulations in human visual cortex.. Neuron.

[54] Kahneman D, Slovic P, Tversky A (1982). Judgment under uncertainty: heuristics and biases.

[55] Gilovich T, Griffin D, Kahneman D (2002). Heuristics and biases: the psychology of intuitive judgment.

