# Supplementary figures and images for: Motor Preparatory Activity in Posterior Parietal Cortex is Modulated by Subjective Absolute Value
Source: PLoS Biol. 2010 Aug 3;8(8):e1000444. doi: 10.1371/journal.pbio.1000444 (PMC2914636; doi:10.1371/journal.pbio.1000444)

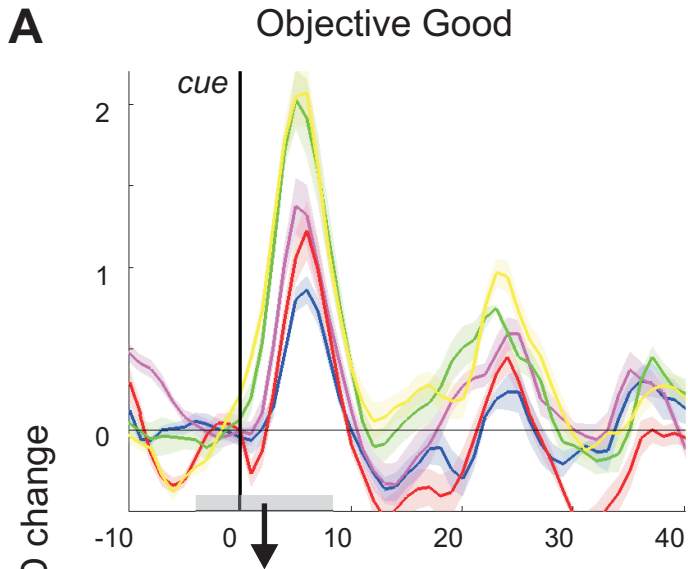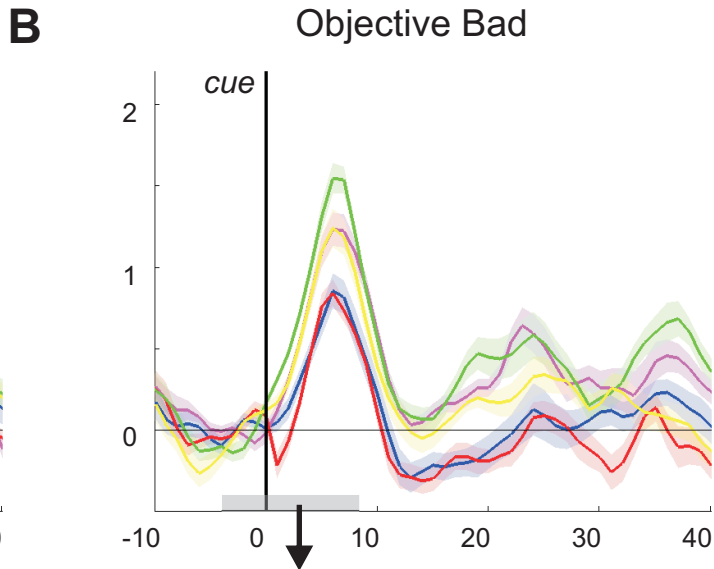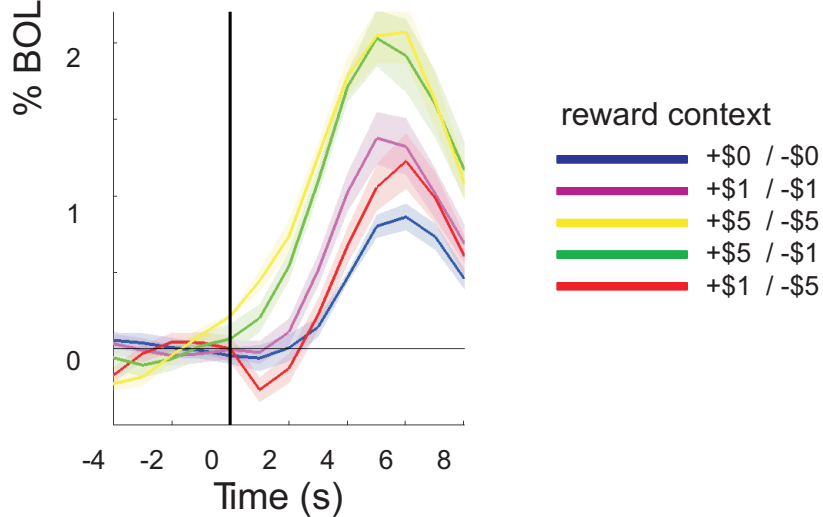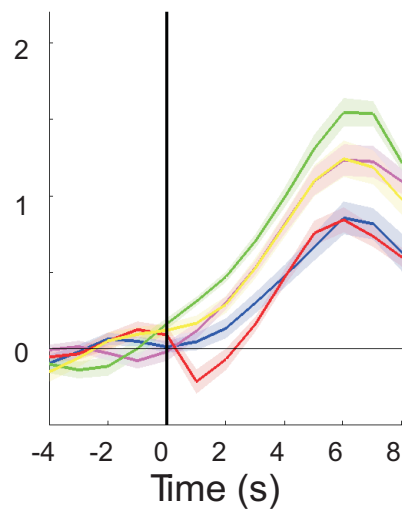

Supplement: Figure S1 — BOLD time-courses of the left dorsal striatum for objective good and bad subjects. The time-course over the entire trial duration is presented on top; two corresponding graphs that zoom in on the cue-related response (0 s denoting onset of gain-loss context cue, black lines) are depicted below. For the respective time-courses according to the subjective performance grouping, please refer to Figure S2. (0.08 MB PDF) [file pbio.1000444.s001.pdf]

# Subjective Good

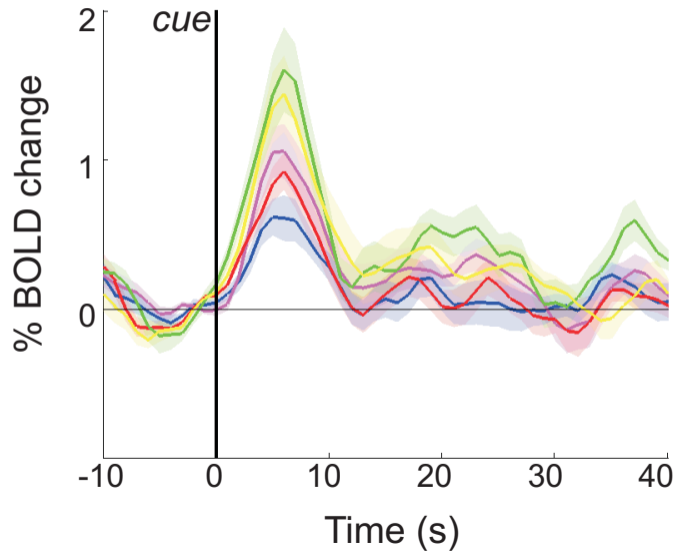

# Subjective Bad

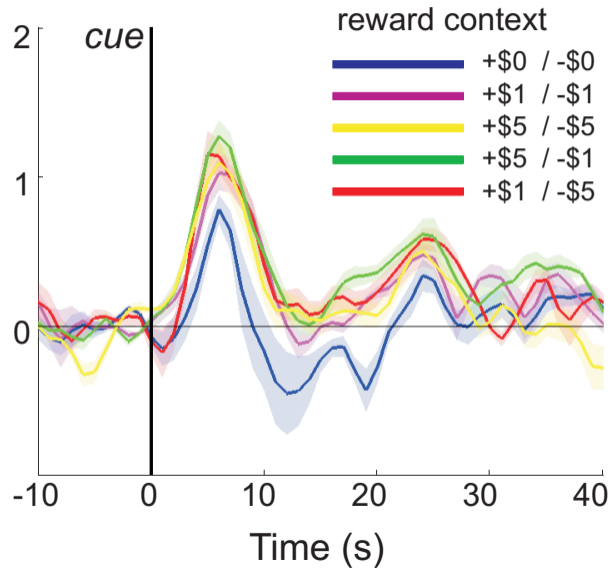

Supplement: Figure S2 — Dorsal striatal BOLD signal time-courses for subjective good and bad subjects. The time-course over the entire trial duration is presented, with black lines indicating the onset of gain-loss context cue presentation. Note that unlike in Figure S1, which was divided on the basis of the objective performance, subjective grouping led to a larger overall variance (error bars). (0.05 MB PDF) [file pbio.1000444.s002.pdf]

## Orbitofrontal cortex (OFC)

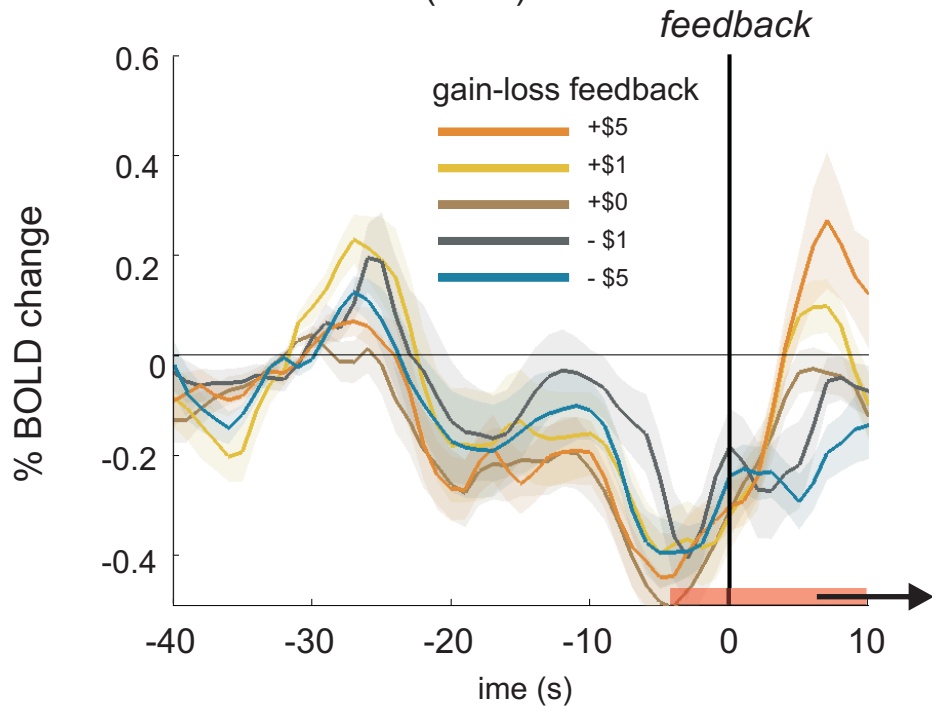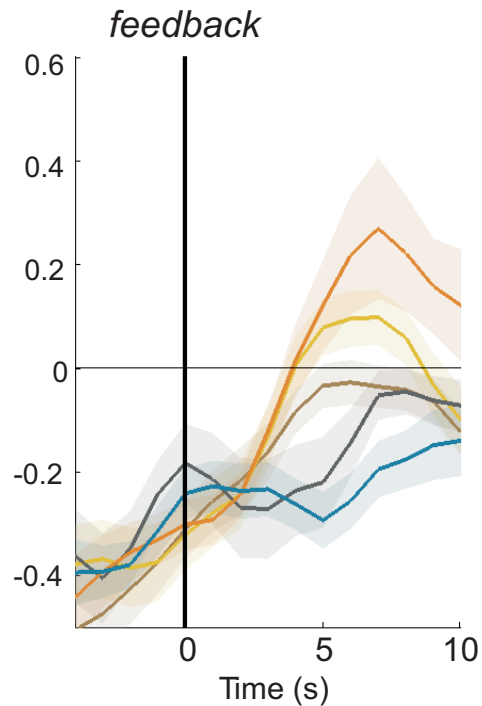

Supplement: Figure S3 — Orbitofrontal cortex BOLD signal time-courses. The upper panel depicts time-courses for the entire trial duration; below a graph that zooms in on the feedback-related response is shown. Black lines at time 0 s correspond to the onset of the feedback information. (0.04 MB PDF) [file pbio.1000444.s003.pdf]
