# Supplementary material for: Motor Preparatory Activity in Posterior Parietal Cortex is Modulated by Subjective Absolute Value
Source: PLoS Biol. 2010 Aug 3;8(8):e1000444. doi: 10.1371/journal.pbio.1000444 (PMC2914636; doi:10.1371/journal.pbio.1000444)
Supplement: Table S1 — Brain regions exhibiting a significant contribution to the delay period. All areas that were considered in our ROI analysis are in italics (p < 0.05 corrected at cluster level; k > 5 voxels; threshold at voxel-level: p < 0.01 FWE-corrected). (0.01 MB PDF) [file pbio.1000444.s004.pdf]

Region	*MNI Coordinates [mm]*			*Peak*
	x	y	z	*t-value*
SMA	*0*	*6*	*54*	*15.00*
	6	15	48	8.83
Post. IPS, L	*-6*	*-69*	*54*	*14.00*
R	*9*	*-69*	*54*	*13.99*
SPL, L	*-18*	*-72*	*54*	*13.06*
PMd, L	*-33*	*-9*	*60*	*10.82*
Occipital, Middle R	30	-66	33	10.28
Occipital, Superior R	27	-78	42	10.27
Ant. IPS, L	*-39*	*-33*	*45*	*9.76*
	-30	-57	48	9.43
	-33	-45	48	8.42
Ant. IPS, R	33	-48	42	8.54
PMd, R	21	0	63	9.81
	30	-6	57	9.00
Ant. Insula, R	33	21	0	9.81
Ant. Insula, L	-30	24	0	8.61

Supplemental Table S1: Brain regions exhibiting a significant contribution *($p<0.05$ corrected at cluster level*; *$k>5$ voxels; threshold at voxel-level: $p<0.01$ FWE-corrected)* to the delay period. All areas that were considered in our ROI analysis are in *italics*.
