# Supplementary material for: Motor Preparatory Activity in Posterior Parietal Cortex is Modulated by Subjective Absolute Value
Source: PLoS Biol. 2010 Aug 3;8(8):e1000444. doi: 10.1371/journal.pbio.1000444 (PMC2914636; doi:10.1371/journal.pbio.1000444)
Supplement: Table S2 — Average R2-values of the linear regression between explanatory models and individual subject's beta estimates for different reward contexts. Significant differences in R2-values of different models are indicated by “X.” Separate tables are provided for all ROIs that showed a significant difference in R2-values across models, namely the left and right posterior IPS (pIPS l & pIPS r) and the SMA. The table for SPL is provided in the main manuscript (Figure 5D). (0.03 MB PDF) [file pbio.1000444.s005.pdf]

**pIPS l**	**… Obj. Value**	**… Subj. Value**	**… Obj. Stakes**	**… Subj. Stakes**	**… Obj. Abs. Value**	**… Subj. Abs. Value**	**… Subj. Moti-vation**
**Obj. Value > …**		-	-	-	-	-	-
**Subj. Value > …**	X		-	-	-	-	-
**Obj. Stakes > …**	X	-		-	-	-	-
**Subj. Stakes > …**	X	-	-		-	-	-
**Obj. Abs. Value > …**	X	-	-	-		-	-
**Subj. Abs. Value > …**	X	X	X	-	X		-
**Subj. Motivation > …**	X	-	-	-	-	-	
**R^2-Values (+/- SEM)**	0.21 (+/-0.05)	0.37 (+/-0.06)	0.36 (+/-0.07)	0.45 (+/-0.08)	0.35 (+/-0.07)	**0.51 (+/-0.08)**	0.44 (+/-0.07)

**pIPS r**	**… Obj. Value**	**… Subj. Value**	**… Obj. Stakes**	**… Subj. Stakes**	**… Obj. Abs. Value**	**… Subj. Abs. Value**	**… Subj. Moti-vation**
**Obj. Value > …**		-	-	-	-	-	-
**Subj. Value > …**	X		-	-	-	-	-
**Obj. Stakes > …**	X	-		-	-	-	-
**Subj. Stakes > …**	X	-	-		-	-	-
**Obj. Abs. Value > …**	X	-	-	-		-	-
**Subj. Abs. Value > …**	X	X	X	-	X		-
**Subj. Motivation > …**	X	-	-	-	-	-	
**R^2-Values (+/- SEM)**	0.24 (+/-0.06)	0.39 (+/-0.06)	0.37 (+/-0.07)	0.45 (+/-0.08)	0.38 (+/-0.07)	**0.52 (+/-0.08)**	0.45 (+/-0.08)

SMA	… Obj. Value	… Subj. Value	… Obj. Stakes	… Subj. Stakes	… Obj. Abs. Value	… Subj. Abs. Value	… Subj. Moti-vation
Obj. Value > …		-	-	-	-	-	-
Subj. Value > …	-		-	-	-	-	-
Obj. Stakes > …	X	-		-	-	-	-
Subj. Stakes > …	X	X	-		-	-	-
Obj. Abs. Value > …	X	X	-	-		-	-
Subj. Abs. Value > …	X	X	-	-	-		-
Subj. Motivation > …	X	-	-	-	-	-	
**R^2-Values (+/- SEM)**	0.23 (+/-0.06)	0.29 (+/-0.06)	0.40 (+/-0.07)	0.43 (+/-0.07)	0.45 (+/-0.06)	**0.46 (+/-0.07)**	0.39 (+/-0.07)

Supplemental Table S2: The tables depict the average R^2-values of the linear regression between the different explanatory models and individual subject's beta estimates for different reward contexts. Significant differences in R^2-values of different models are indicated by 'X'. Separate tables are provided for all ROIs that showed a significant difference in R^2-values across models, namely the left and right posterior IPS (pIPS l & pIPS r) and the SMA. The table for SPL is provided in the main manuscript (Figure 5D).
