# Supplementary material for: Motor Preparatory Activity in Posterior Parietal Cortex is Modulated by Subjective Absolute Value
Source: PLoS Biol. 2010 Aug 3;8(8):e1000444. doi: 10.1371/journal.pbio.1000444 (PMC2914636; doi:10.1371/journal.pbio.1000444)
Supplement: Table S3 — Parametric modulation of the delay-related BOLD-signal. Only regions that exhibit a significant (p < 0.05 corrected at cluster level; k > 5 voxels; threshold at voxel-level: p < 0.05 FDR-corrected) correlation with our parametric modulators are listed. (0.01 MB PDF) [file pbio.1000444.s006.pdf]

Region	*MNI Coordinates [mm]* x	y	z	*Peak* *t-stat*
Delay: Absolute value, subjective performance				
SPL, L	-15	-72	54	5.13
R	12	-75	51	7.72
Post. IPS, L	-3	-75	48	4.70
R	12	-75	54	6.89
Ant. Insula, R	30	24	3	5.31
Ant. IPS, R	45	-45	39	5.22
SMA	6	6	54	4.99
Delay: Gains				
Frontal Operculum	48	6	24	5.02
Precentral Gyrus	54	0	30	4.21

Supplemental Table S3: Parametric modulation of the delay-related BOLD-signal. Only regions that exhibit a significant *(p<0.05 corrected at cluster leve*; *k>5 voxels; threshold at voxel-level: p<0.05 FDR-corrected)* correlation with our parametric modulators are listed.
