# Supplementary material for: Motor Preparatory Activity in Posterior Parietal Cortex is Modulated by Subjective Absolute Value
Source: PLoS Biol. 2010 Aug 3;8(8):e1000444. doi: 10.1371/journal.pbio.1000444 (PMC2914636; doi:10.1371/journal.pbio.1000444)
Supplement: Table S4 — Regions that exhibited a significant difference between models. Second-level analyses were based on individual subject GLMs including a single explanatory model (p < 0.05 corrected at cluster level p < 0.05; k > 5 voxels; threshold at voxel-level: p < 0.05 FDR-corrected; inclusive mask for delay period activity at p < 0.01 FWE-corrected; k > 5 voxels). (0.02 MB PDF) [file pbio.1000444.s007.pdf]

	… Objective Value			… Subjective Value	… Objective Stakes			… Subjective Stakes			… Objective Abs. Value			… Subjective Abs. Value
**Objective Value > …**														
**Subjective Value > …**														
**Objective Stakes > …**														
**Subjective Stakes > …**														
**Objective Abs. Value > …**														
**Subjective Abs. Value > …**	**SPL, R**	12 -66 48	4.67		**SPL, L**	-15 -66 63	6.12	**Post. IPS, R**	6 -78 45	4.73	**Post. IPS, R**	6 -78 45		
	**Post. IPS, R**	9 -72 54	4.43			-24 -66 60	4.78	**SPL, L**	-9 -69 60	4.65	**SPL, R**	9 -72 54		
	**Post .IPS, L**	-3 -69 48	4.03			-9 -57 69	4.45							
	**Ant. IPS, L**	-36 -54 42	3.82		**Post. IPS, R**	6 -78 45	4.78							
	**SPL, L**	-9 -69 42	3.79			15 -78 51	4.51							
	**PO, R**	33 -75 36	3.76		**PO, R**	24 -84 39	4.21							
					**Post. IPS, L**	-9 -75 51	3.99							
					**SPL, L**	-39 -60 54	3.79							
					**SPL, R**	30 -57 66	3.76							
						6 -66 60	3.69							

Supplemental Table S4: Regions that exhibited a significant difference between models *(p<0.05 corrected at cluster level p<0.05*; *k>5 voxels; threshold at voxel-level: p<0.05 FDR-corrected; inclusive mask for delay period activity at p< 0.01 FWE-corrected; k>5 voxels).* Second level analyses were based on individual subject GLMs including a single explanatory model.
