# Supplementary material for: Motor Preparatory Activity in Posterior Parietal Cortex is Modulated by Subjective Absolute Value
Source: PLoS Biol. 2010 Aug 3;8(8):e1000444. doi: 10.1371/journal.pbio.1000444 (PMC2914636; doi:10.1371/journal.pbio.1000444)
Supplement: Table S5 — Parametric modulation of the cue-related BOLD-signal. Only regions that exhibit a significant (p < 0.05 corrected at cluster level; k > 5 voxels; threshold at voxel-level: p < 0.05 FDR-corrected) correlation with our parametric modulators are listed. (0.01 MB PDF) [file pbio.1000444.s008.pdf]

Region	*MNI Coordinates [mm]*			*Peak*
	x	y	z	*t-stat*
Cue: Value, objective performance				
Caudate, L	-9	18	0	4.59
R	6	15	0	5.20
Thalamus, L	-12	-18	18	6.12
R	9	-6	18	5.38
Hippocampus, R	33	-39	6	5.36
Calcarine, L	-27	-63	18	6.07
R	33	-60	12	5.99
Inferior Frontal Gyrus, L	-45	15	33	5.63
Cue: Absolute value, subjective performance				
Caudate, L	-9	21	0	3.92
R	18	21	12	5.03
	21	6	18	4.75
	21	9	21	4.70
	18	24	0	4.15
Thalamus, R	12	-15	18	5.61
Cuneus, L	-6	-90	24	6.63
Ant. IPS, L	-27	-60	42	4.95
Middle Occipital, L	30	-72	24	4.93
Cue: Risk				
Calcarine, L	-9	-72	15	4.88
Cue: Gains				
Post. Cingulate / Precuneus, L	-9	-42	3	5.34

Supplemental Table S5: Parametric modulation of the cue-related BOLD-signal. Only regions that exhibit a significant *($p<0.05$ corrected at cluster level*; *$k>5$ voxels; threshold at voxel-level: $p<0.05$ FDR-corrected)* correlation with our parametric modulators are listed.
