# Supplementary material for: Motor Preparatory Activity in Posterior Parietal Cortex is Modulated by Subjective Absolute Value
Source: PLoS Biol. 2010 Aug 3;8(8):e1000444. doi: 10.1371/journal.pbio.1000444 (PMC2914636; doi:10.1371/journal.pbio.1000444)
Supplement: Table S6 — Parametric modulation of the outcome-related BOLD-signal. Only regions that exhibit a significant (p < 0.05 corrected at cluster level; k > 5 voxels; threshold at voxel-level: p < 0.001 uncorrected) correlation with our parametric modulators are listed. (0.01 MB PDF) [file pbio.1000444.s009.pdf]

Region	*MNI Coordinates [mm]*			*Peak*
	x	y	z	*t-stat*
Outcome: Reward > Punishment				
Ventral Striatum, L	-9	6	-3	6.10
	-6	9	0	5.75
R	6	6	-3	5.16
Caudate, L	-6	6	6	5.27
Putamen, L	-12	9	-6	5.67
R	15	9	-6	6.39
Insula-Caudal Orbitofrontal, L	-36	18	-15	7.22
Ant. Insula, L	-30	18	6	4.95
Inferior Parietal, L	-51	-54	51	6.33
R	51	-54	48	5.65
Ant. Cingulate, L	0	33	9	5.50
R	12	39	21	5.75
Inferior Frontal Gyrus, R	36	27	27	5.25
Orbitofrontal, L	0	48	-6	6.46
Outcome: Large Reward($5) > Small Reward($1) > Small Punishment(-$1) > Large Punishment(-$5)				
Middle Frontal Gyrus, L	-48	12	45	5.67
Inf. Frontal Gyrus, R	30	33	0	4.47
Orbitofrontal, R	6	51	-6	4.29
Caudate, R	-3	18	0	3.80
Outcome: Punishment > Reward				
PMd, L	-27	-15	69	4.16
R	24	-24	72	3.80
Postcentral Gyrus, R	33	-36	69	4.50

Supplemental Table S6: Parametric modulation of the outcome-related BOLD-signal. Only regions that exhibit a significant *(p<0.05 corrected at cluster level*; *k>5 voxels; threshold at voxel-level: p<0.001 uncorrected)* correlation with our parametric modulators are listed.
