# Supplementary material for: Motor Preparatory Activity in Posterior Parietal Cortex is Modulated by Subjective Absolute Value
Source: PLoS Biol. 2010 Aug 3;8(8):e1000444. doi: 10.1371/journal.pbio.1000444 (PMC2914636; doi:10.1371/journal.pbio.1000444)
Supplement: Text S1 — Supplemental results and discussion. We provide additional results about fMRI responses elicited by the contextual gain-loss cues and also describe fMRI responses to the reward outcome. Finally, we discuss the involvement of canonical reward structures in the encoding of the reward context. (0.06 MB DOC) [file pbio.1000444.s010.doc]

**SUPPLEMENTAL MATERIAL**

***Supplemental Results***

*Neural Responses to Spatial and Gain-Loss Contextual Cues*

As the contextual cue supplies gain-loss information, areas engaged in processing this context information are potentially necessary for modulating action preparatory activity. Regions exhibiting a significant response to the cue presentation (p(FWE)<0.01, k>5) encompassed subcortical structures including the thalamus and the striatum; and cortical clusters bilaterally in precuneus, supplementary motor area, SPL, dorsal premotor cortex, middle occipital gyrus, posterior cingulate, and in the left lingual and fusiform gyri.

For 2^nd^ level GLMs predicated upon stakes and value (either rooted in objective or subjective performance estimates) or predicated upon subjective preference and motivation, a small subset of the cue-activated brain regions —the thalamus, caudate, and posterior cingulate/precuneus—revealed a significant parametric modulation due to the gain-loss context. Presumably, those areas not parametrically modulated are more involved in general sensory or mnemonic processing of the cue, rather than in encoding specific contingencies signified by the gain-loss context. Consistent with previous findings [5,6], thalamic and caudate cue-related activity demonstrated a value-related modulation of BOLD-activity (Supplemental Table S5). Caudate BOLD time-courses for the objective good and the objective bad group are shown in supplemental figure S1. In the time-courses presented on the left, the good performance “weights” the gains of the gain-loss contexts, leading to high signal amplitudes in both high-gain contexts (+$5/-$5, +$5/-$1), and intermediate amplitudes in low-gain contexts (+$1/-$1, +$1/-$5, both higher than $0/-0). Conversely, averaged over the objective bad subjects, where performance now “weights” the losses more, high-gain contexts showed overall lower signal amplitudes, the amplitude in the high-gain/high-loss context was smaller than in the high-gain/low-loss context, and the amplitude of the low-gain/high-loss context did not differ from the $0/-0 condition. For direct comparison with signals in motor preparatory ROIs, caudate time-courses for *subjective* good and bad groups are shown in Supplemental Figure S2. Besides cue-responsive ROIs in caudate, additional clusters, while not showing a main effect of the cue, demonstrated robust contextual modulation during the cue epoch. A subset of these clusters showed modulation consistent with objective value, as with the cue-activated caudate ROIs. However, the remaining clusters displayed BOLD patterns resonant with subjective absolute value. Peak voxels of absolute value-related modulation in the caudate generally lay more dorsolaterally than voxels with value-modulated activity (for a comprehensive list of clusters showing modulation of the cue epoch, see Supplemental Table S5).

To discern other reward-related areas that may exhibit context-related responses, the statistical threshold at the voxel level was eased (p<0.01 uncorrected); additional voxels in the orbitofrontal cortex then showed similar objective value-related modulation (right [6 51 -6; 3 54 -12 mm x y z, MNI-space], *t* = 3.48, 3.50 for parametric modulation).

*Neural Responses to Outcome*

Areas which process feedback about events carry information important for the comparison of actual vs. predicted outcomes, and for the development of future expectations. In this experiment, feedback about monetary gains/losses at the end of each trial elicited significant cortical and subcortical activation (p<0.05 corrected at cluster-level; k>5 voxels; threshold at voxel-level: p<0.001 uncorrected; compare Supplemental Table S6). In accordance with prior studies, bilateral ventral striatum, bilateral putamen, and caudate showed a greater BOLD response to gains as compared to losses [12,36]. Regions within the right inferior frontal gyrus and bilateral anterior cingulate, inferior parietal, and medial prefrontal displayed similar reward-related signals. Additionally, BOLD changes scaling positively with the magnitude of the rewards and inversely with the magnitude of punishments on each trial were observed in the right medial orbitofrontal cortex and the right caudate (see Supplemental Figure S3) [10,12]. Punishments produced greater BOLD responses than did rewarding outcomes in precentral (PMd) and postcentral gyri. No voxels demonstrated activity positively correlated with the magnitude of punishment.

***Supplemental Discussion***

*Involvement of Canonical Reward Structures in Encoding Reward Context*

Among canonical reward structures, the caudate exhibited the most significant modulation in response to the reward-predicting/spatial cue. Comparable striatal evaluation of reward-predicting cues has been documented in human and nonhuman primates [5,12,39,56-59]. The dorsal striatum, particularly the caudate, plays a critical role in establishing associations between an (goal-directed) action and its outcome and the current value of the outcome [23,25,42,60-65], underscoring its involvement with feedback-sensitive goal-directed actions [66,67]. However, previous experiments that sought to elucidate factors influencing the valuation of action have done so by manipulating predominantly stimulus-outcome associations; and factors, such as risk, uncertainty, probability and mean (expected) value were all externally controlled (e.g. varied by the experimenter). Extending these findings, we show that reward structures also incorporate an estimation of response completion and outcome, the likelihood of which is governed by the subject’s performance. Interestingly, in our paradigm where objective and subjective estimates of performance diverged drastically, striatal computations of value mostly relied upon the subjects’ objective performance.

The ventral and dorsal striatum receive dense dopaminergic innervation, which has been proposed to carry a prediction error signal [11,68,69]. This signal, which may veridically reflect the error between actual and predicted occurrences, might in turn be exploited by the striatum, underlying its role in the learning of selection preferences on the basis of obtained rewards and punishments [16,70-72].

In addition to a value-encoding population, a subset of voxels in the dorsal striatum, though not exhibiting significant responses to the cue presentation irrespective of reward context, showed significant absolute value related modulation during this cue period. Tonically active striatal neurons, which respond to both positive and negative predictive cues but not neutral stimuli [73], could conceivably account for our observed BOLD signal reflecting absolute value modulation but no main cue effect. Furthermore, voxels demonstrating absolute-value-related BOLD activity tended to cluster more dorsolaterally in the caudate than those showing value modulation (see Supplemental Table S6).

Anatomical examinations of basal ganglia connectivity expose several parallel corticostriatal loops subserving different functions [74,75]: ventromedial striatal areas, to which more limbic roles have been ascribed, receive projections from orbitofrontal and anterior cingulate cortices [75]; conversely, afferents from association cortices, including dorsolateral prefrontal and posterior parietal cortex, terminate in more dorsolateral regions of the striatum. In this study, orbitofrontal cortex activity resembled that of the ventromedial striatum, whereas more central and dorsolateral areas of the caudate displayed modulation similar to that of the motor-preparatory areas (including parietal cortices). Our data thus poses the question of whether distinct basal ganglia-cortical loops differentially process and utilize cue/object-related information about reward context on the one hand and in order to bias cortical action preparation on the other hand.

***Supplemental references***

56. Kawagoe R, Takikawa Y, Hikosaka O (1998) Expectation of reward modulates cognitive signals in the basal ganglia. Nat Neurosci 1: 411- 416.

57. Cromwell HC, Schultz W (2003) Effects of expectations for different reward magnitudes on neuronal activity in primate striatum. J Neurophysiol 89, 5: 2823-2838.

58. Apicella P, Ljungberg T, Scarnati E, Schultz W (1991) Responses to reward in monkey dorsal and ventral striatum. Exp Brain Res 85, 3: 491-500.

59. Gold J (2003) Linking reward expectation to behavior in the basal ganglia. Trends in Neurosciences 26, 1: 12-14

60. Dickinson A, Balleine BW (1994) Motivational control of goal-directed action. Animal Learning & Behavior 22: 1-18.

61. Balleine BW, Dickinson A. (1998) Goal-directed instrumental action: contingency and absolute value learning and their cortical substrates. Neuropharmacology 37, 4-5: 407-419.

62. Haruno M, Kuroda T, Doya K, Toyama K, Kimura M, et al. (2004) A neural correlate of reward-based behavioral learning in caudate nucleus: a functional magnetic resonance imaging study of a stochastic decision task. J. Neurosci 24: 1660 -1665.

63. Tremblay L, Hollerman JR, Schultz W (1998) Modifications of reward expectation-related neuronal activity during learning in primate striatum. J Neurophysiol 80: 964 -977.

64. Kawagoe R, Takikawa Y, Hikosaka O (2004) Reward-predicting activity of dopamine and caudate neurons--a possible mechanism of motivational control of saccadic eye movement. J Neurophysiol 91, 2: 1013-1024.

65. Shidara M, Aigner TG, Richmond BJ (1998) Neuronal signals in the monkey ventral striatum related to progress through a predictable series of trials. J Neurosci 18: 2613-2625.

66. Haruno M, Kawato M (2006) Different neural correlates of reward expectation and reward expectation error in the putamen and caudate nucleus during stimulus-action-reward association learning. J Neurophysiol 95, 2: 948-959.

67. Levy R, Dubois B (2006) Apathy and the functional anatomy of the prefrontal cortex-basal ganglia circuits. Cereb Cortex 16, 7: 916-928.

68. Montague PR, Dayan P, Sejnowski TJ (1996) A framework for mesencephalic dopamine systems based on predictive Hebbian learning. J Neurosci 16, 5: 1936-1947.

69. Schultz W (1997) Dopamine neurons and their role in reward mechanisms. Curr Opin Neurobiol 7, 2: 191-197.

70. McClure SM, York MK, Montague PR (2004) The Neural Substrates of Reward Processing in Humans: The Modern Role of fMRI. Neuroscientist 10, 3: 260-268.

71. Tobler PN, O'Doherty JP, Dolan RJ, Schultz W (2006) Human neural learning depends on reward prediction errors in the blocking paradigm. J Neurophysiol 95, 1: 301-310.

72. Hollerman JR, Tremblay L, Schultz W (1998) Influence of reward expectation on behavior-related neuronal activity in primate striatum. J Neurophysiol 80: 947-963.

73. Ravel S, Legallet E, Apicella P (2003) Responses of tonically active neurons in the monkey striatum discriminate between motivationally opposing stimuli. J Neurosci 23, 24: 8489-8497.

74. Nakano K (2000) Neural circuits and topographic organization of the basal ganglia and related regions. Brain Dev. 22, Suppl 1: S5-S16.

75. Selemon LD, Goldman-Rakic PS (1988) Common cortical and subcortical targets of the dorsolateral prefrontal and posterior parietal cortices in the rhesus monkey: evidence for a distributed neural network subserving spatially guided behavior. J Neurosci 8, 11: 4049-4068.
